# Design, Synthesis and Biological Evaluation of Benzohydrazide Derivatives Containing Dihydropyrazoles as Potential EGFR Kinase Inhibitors

**DOI:** 10.3390/molecules21081012

**Published:** 2016-08-03

**Authors:** Hai-Chao Wang, Xiao-Qiang Yan, Tian-Long Yan, Hong-Xia Li, Zhong-Chang Wang, Hai-Liang Zhu

**Affiliations:** 1 College of Biology and Food Engineering, Suzhou University, Suzhou 234000, China; wanghc0823@163.com (H.-C.W.); hxl96hx@163.com (H.-X.L.); 2 State Key Laboratory of Pharmaceutical Biotechnology, Nanjing University, Nanjing 210093, China; 18120176083@163.com (X.-Q.Y.); ytlnju@163.com (T.-L.Y.)

**Keywords:** benzohydrazide derivatives, EGFR inhibitor, antiproliferative activity, cytotoxicity, molecular docking

## Abstract

A series of novel benzohydrazide derivatives containing dihydropyrazoles have been synthesized as potential epidermal growth factor receptor (EGFR) kinase inhibitors and their biological activities as potential antiproliferative agents have been evaluated. Among these compounds, compound **H20** exhibited the most potent antiproliferative activity against four cancer cell line variants (A549, MCF-7, HeLa, HepG2) with IC_50_ values of 0.46, 0.29, 0.15 and 0.21 μM respectively, which showed the most potent EGFR inhibition activities (IC_50_ = 0.08 μM for EGFR). Molecular modeling simulation studies were performed in order to predict the biological activity and activity relationship (SAR) of these benzohydrazide derivatives. These results suggested that compound **H20** may be a promising anticancer agent.

## 1. Introduction

Cancer has become one of the most serious diseases which represents a great threat to human health around the world [1]. Although there are currently a large number of anticancer drugs, more and more people continue to die of cancer [2]. Epidermal growth factor receptor (EGFR) plays important roles in human cancer. As a member of the HER family, EGFR is a tyrosine kinase receptor which plays an essential role in normal cell growth and differentiation, and is involved in tumor proliferation and survival [3,4]. The HER family comprises four members: EGFR (HER1/ErbB-1), ErbB-2 (HER2/neu), ErbB-3 (HER3), and ErbB-4 (HER4) [5]. EGFR is one of the most important targets in current cancer research and its over-expression or abnormal activation often causes cell malignant transformation [6]. The over-expression of EGFR has been observed in many solid tumors, such as colon [7], ovarian [8], breast and non-small cell lung cancer (NSCLC) [9]. Therefore, EGFR inhibition has been developed as one of the most efficient strategies for cancer therapy.

**Figure 1:**
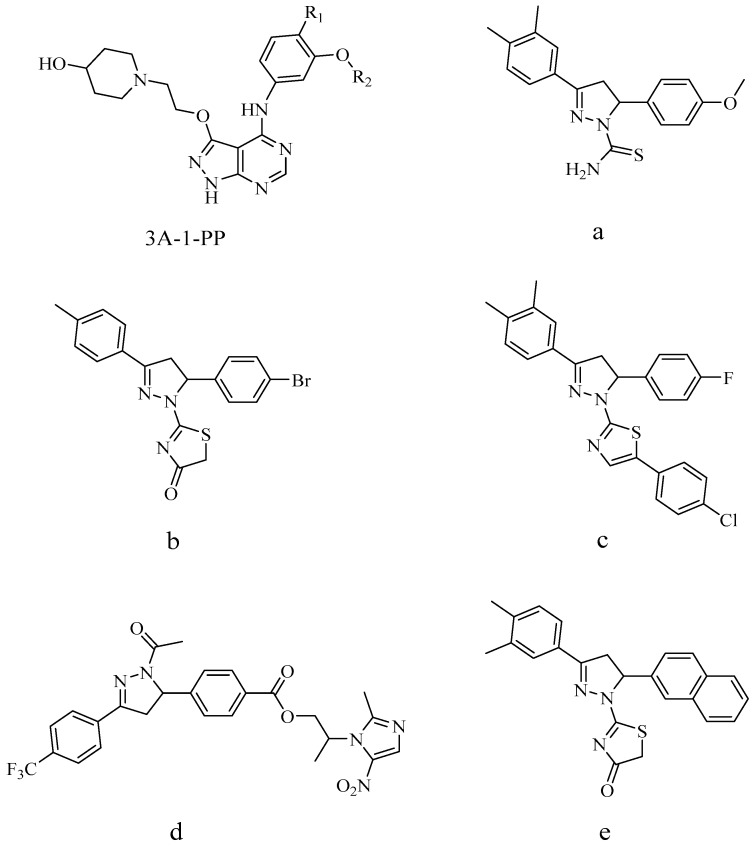
Chemical structures of some reported compounds.

Dihydropyrazole, a small bioactive molecule, is a prominent structural motif found in numerous pharmaceutically active compounds. Some representatives had been demonstrated to have important biological activities such as antiviral/antitumor [10], antibacterial [11], fungistatic [12], antihyperglycemic activity [13], antimalarial [14] and antitubercular effects [15]. Biological evaluation indicated that some of the synthesized compounds were potent inhibitors of EGFR. Some novel compounds containing the dihydropyrazole skeletons were reported as potent anticancer agents against EGFR tyrosine kinase. It had been reported that anticancer compounds such as 3-alkoxy-1*H*-pyrazolo[3,4-*d*]pyrimidines analogues (3A-1-PP, Figure 1) showed potent EGFR kinase inhibitory activity, with IC_50_ values reaching single digit nanomolar values [16]. Compounds **a**, **b**, **c** and **d** (Figure 1) displayed the most potent EGFR inhibitory activity, with IC_50_ values of 0.07, 0.24, 0.06 and 0.26 μM respectively, which were comparable to the positive control erlotinib (IC_50_ = 0.03 μM) [17,18,19,20]. Compound **e** containing the dihydropyrazole and naphthalene ring showed the highest inhibitory EGFR activity (IC_50_ = 0.12 μM) [21].

Some naphthalene derivatives have been reported as potent microtubule inhibitors, apoptosis inducers, glutamamide analogues or P-glycoprotein inhibitors [22,23]. On the other hand, the naphthalene ring can be recognized in various biologically active compounds with clinical applications. Naftifine and terbinafine, allylamine antifungal agents, are widely used for the treatment of fungal infections [24,25,26]. Propranolol is an anti-angina drug, duloxetine is used for the treatment of depression, nafimidone and its oxime ester derivative can be further developed for the treatment of epilepsy [27]. Therefore, preparation and extensive biological evaluations on naphthalene derivatives have continuously attracted our attention [28]. Besides, benzohydrazides are reported to possess a wide variety of biological activities like antiglycation [29], antioxidant [30], antileishmanial [22], antibacterial [31], antifungal [23], antitumor [32] and anticonvulsant [24]. Benzothiazole Schiff bases with diverse biological activities have also been reported [26].

The three combined substructures—the dihydropyrazole along with the naphthalene ring and benzohydrazides—might exhibit synergistic anticancer effects. All of these facts encouraged us to integrate these three moieties and screen new benzohydrazide derivatives containing dihydropyrazoles as potential EGFR inhibitory agents. Herein we disclose the design and synthesis of some novel benzohydrazide derivatives containing dihydropyrazoles as potential EGFR kinase inhibitors, and their antitumor activity against the A549 (human lung cancer), MCF-7 (human breast cancer), HeLa (human cervical cancer) and HepG2 (human hepatocellular cancer) cancer cell lines.

## 2. Results and Discussion

### 2.1. Chemistry

**Scheme 1:**
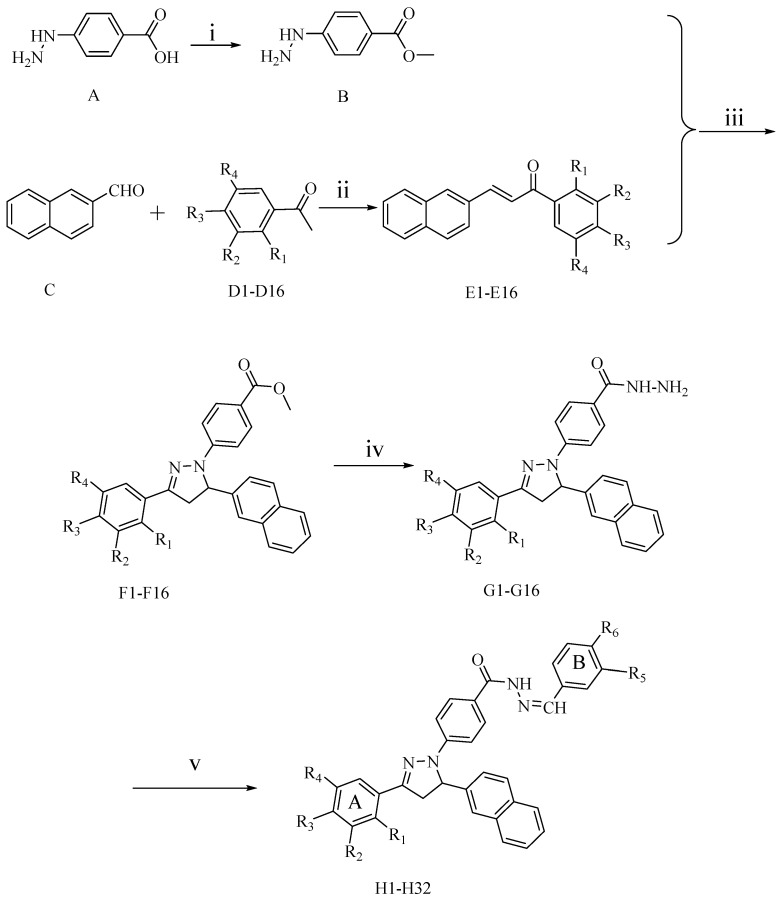
The synthetic routes to compounds **H1**–**H32**. *Reagents and conditions:* (**i**) MeOH, SOCl_2_, room temperature, 20–24 h; (**ii**) EtOH, KOH, room temperature, 6–8 h; (**iii**) EtOH, HAc, 80 °C, 20–24 h; (**iv**) MeOH, N_2_H_4_·H_2_O (80%), DMF, 70 °C, 20–24 h; (**v**) RCHO, EtOH, HOAc, room temperature, 6–8 h.

A series of novel benzohydrazide derivatives containing dihydropyrazole moieties were synthesized by the routes outlined in Scheme 1. The structures of the compounds are listed in Table 1. Methyl 4-hydrazinylbenzoate (**B**) was synthesized from 4-hydrazinylbenzoic acid in methanol at room temperature. The diverse substituted chalcones **E1**–**E16** were obtained by stirring substituted acetophenones and naphthaldehyde in ethanol at room temperature for 6–8 h. The cyclization different chalcones and methyl 4-hydrazinylbenzoate in refluxing ethanol gives compounds **F1**–**F16**.

Compounds **F1**–**F16** further reacted with excess hydrazine hydrate (80%) to give the requisite intermediate **G1**–**G16**. The desired compounds **H1**–**H32** were generated by the reaction of compounds **G1**–**G16** with substituted benzaldehydes in ethanol for 6–8 h. Purified compounds **H1**–**H32** were finally obtained by chromatography. All synthesized compounds **H1**–**H32** gave satisfactory analytical and spectroscopic data in full accordance with their depicted structures, and other data published in a Chinese patent [33].

### 2.2. Bioassays

#### 2.2.1. Antiproliferative Assay

The antiproliferative activities of the newly synthesized derivatives **H1**–**H32** were evaluated under identical conditions by the MTT assay against four cultured cell lines (A549, MCF-7, HeLa and HepG2) with erlotinib as control. The IC_50_ values of the compounds against these human cancer cells are summarized in Table 2. As shown, the results revealed that all of the target compounds exhibited significant antiproliferative activities, ranging from 0.15 to 100 μM.

Among them, compound **H20**, which had IC_50_ values of 0.46, 0.29, 0.15 and 0.21 μM against A549, MCF-7, HeLa and HepG2, respectively, displayed the most potent antiproliferative activity, comparable to that of the positive control drug erlotinib (with corresponding IC_50_ values of 0.20, 0.12, 0.17 and 0.10 μM). These results suggested that compound **H20** is more potent than the other compounds overall. Modification of substituents such as methyl, halogen, methoxyl, ethyl and ethoxy was performed to explore the structure-activity relationships of these compounds. Compounds bearing one methyl group at the *para*-position in the A ring showed stronger antiproliferative activity (the IC_50_ values of **H4** and **H20**: 0.45 and 0.21 μM against HeLa cells) than those with hydrogen (**H1** and **H17**), fluorine (**H11** and **H27**), chlorine (**H14** and **H30**), bromine (**H16** and **H32**), ethyl (**H5** and **H21**), ethoxy (**H8** and **H24**) and methoxyl (**H6** and **H22**) substituents, in the order of Me > H > Br > Cl > F, Me > OMe and Et > OEt. This means that compounds with electron-donating groups at the *para*-position of the A ring had better antiproliferative activity than those with electron-withdrawing groups. A comparison of the composition on the A ring was as follows: for compounds **H2**–**H4**, **H9**–**H11**, **H13**–**H14**, **H18**–**H20**, **H25**–**H27** and **H29**–**H30**, the potency order of substituent groups on the A ring was found to be *para* > *ortho* > *meta*. Compounds **H9**–**H15** and **H25**–**H31** with halogen groups on the A ring exhibited antiproliferative activities in the order of disubstituted > monosubstituted. Interestingly, compounds **H6** and **H22** with one methoxyl group on the A ring showed stronger antiproliferative activities than compounds **H7** and **H23** with three methoxyl groups, so we concluded that more electron-donating groups could reduce the electron density of the A ring and make it difficult to form *π*-*π* bonds with amino acids containing aromatic groups.

In the case of constant A ring substituents, changes of substituents on the B ring can also affect the activities of these compounds. Contrary to the A ring, derivatives **H17**–**H32** with a methoxyl at the *para*-position displayed better activities than at the *meta*-position (compounds **H1**–**H16**). These indicated that the position of the substituent had an influence on the antitumoral activity.

#### 2.2.2. Kinase Inhibitory Activity

All these newly synthesized compounds were evaluated for their inhibitory activities against EGFR kinases using a solid-phase ELISA assay. The approved EGFR inhibitor drug erlotinib was used as a positive control. The inhibition constants (IC_50_) of the compounds were summarized in Table 3.

The EGFR kinase inhibitory activity results of the tested compounds correlated with the structural relationships (SAR) of their inhibitory effects on the cell proliferation assay. Among the tested compounds, compound **H20** showed potent anticancer activity with IC_50_ of 0.08 μM, which was comparable to the positive control erlotinib (IC_50_ = 0.03 μM). Other tested compounds displayed moderate inhibitory activities, with IC_50_ values ranging from 0.35 μM to 24.58 μM. This suggests that the potent inhibitory effects of the synthetic compounds on the cell proliferation assay were basically related to their kinase inhibitory activities.

#### 2.2.3. Cytotoxicity Test

All the target compounds **H1**–**H32** were evaluated for their toxicity against 293T human kidney epithelial cells at the median cytotoxic concentration (CC_50_) value of the tested compounds determined by the MTT assay. As displayed in Table 4, these compounds were tested at multiple doses to study the viability of 293T cells. Judging from the median cytotoxic concentration (CC_50_) data, all of the tested compounds demonstrated similar cytotoxic activities as erlotinib in vitro against human kidney epithelial 293T cell.

#### 2.2.4. Analysis of Apoptosis

**Figure 2:**
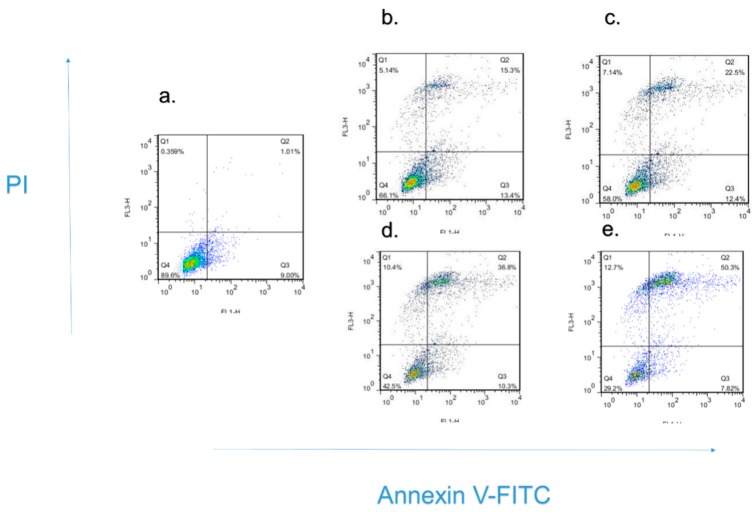
Compound **H20** induced apoptosis in Hela cells with the density of 0, 2.0, 4.0, 6.0, 8.0 μM (**a**–**e**). HeLa cells were treated with for 24 h. Values represent the mean ± S.D, *n* = 3, *p* < 0.05 versus control. The percentage of cells in each part was indicated.

**Figure 3:**
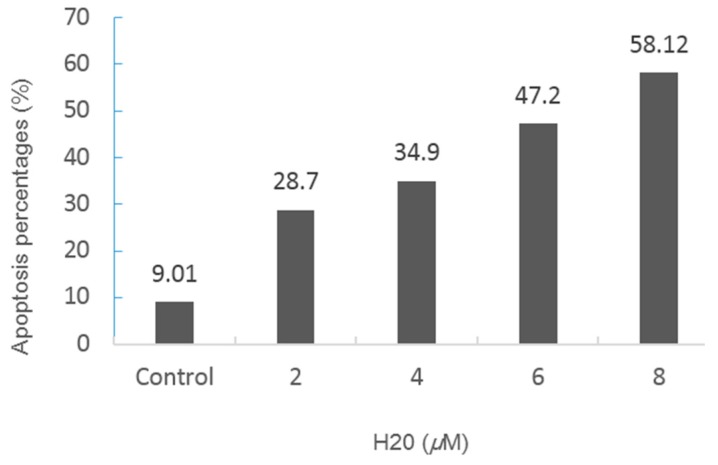
The percentage of early apoptotic cells and late apoptotic cells located in the upper right quadrant. Images were representative of three independent experiments. Values represent the mean ± S.D, *n* = 3, *p* < 0.05 versus control.

To verify whether the inhibition of cell growth of HeLa cell lines was related to cell apoptosis, we decided to use flow cytometry with an Annexin V-FITC/PI Apoptosis Detection Kit to induce the HeLa cell apoptosis with compound **H20**. The uptake of Annexin V-FITC/PI markedly increased, and the uptake of normal cells was significantly decreased in a time-dependent manner. According to the annotated data, the percentage of apoptotic cells was significantly elevated directly and in a dose-dependent manner. The results are shown in Figure 2 and Figure 3. As can be seen, the percentages of cell apoptosis were 9.01%, 28.7%, 34.9%, 47.7%, 58.12%, in response to 0, 2.0, 4.0, 6.0, 8.0 μM concentrations of compound **H20**, respectively. This leads to the conclusion that the percentage of apoptotic cells significantly increased after treatment with high doses of **H20**.

### 2.3. Molecular Docking

**Figure 4:**
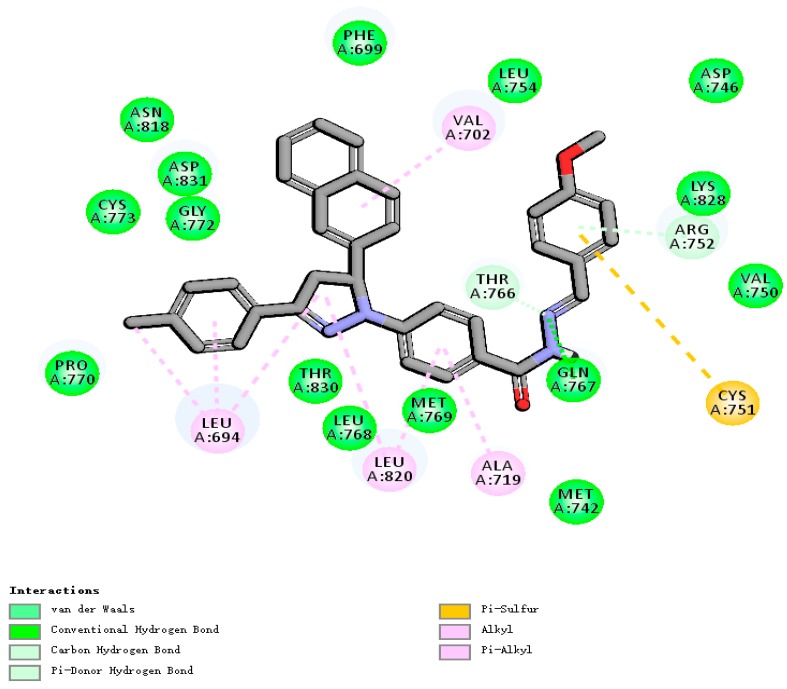
Molecular docking 2D model of the interaction between compound **H20** and EGFR enzyme: for clarity, only interacting residues are displayed.

**Figure 5:**
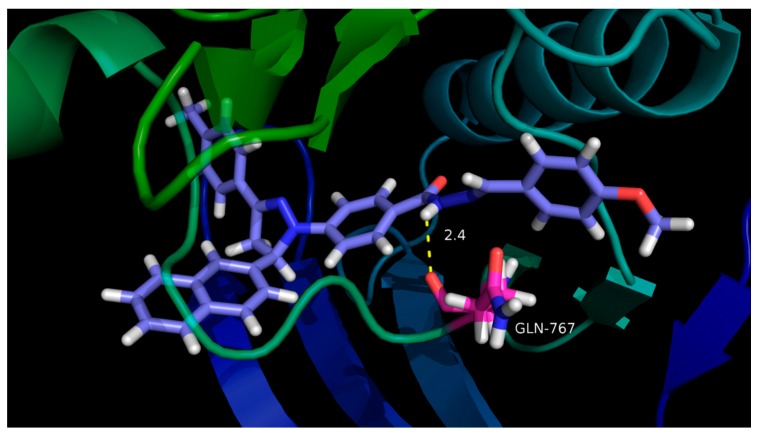
Molecular docking 3D modeling of compound **H20** with the EGFR enzyme: for clarity, only interacting residues are displayed.

To gain better understanding of the potency of the 32 compounds and guide further SAR studies, we examined the interaction of these compounds with EGFR (PDB code: 1M17) by molecular docking. A simulation of binding between the compounds and the ATP binding sites in EGFR was performed. All docking runs used the DS 4.5 software (Discovery Studio 4.5, Accelrys, Co. Ltd., Beijing, China). The obtained results, presented in Figure 4 and Figure 5, show the optimal binding mode of compound **H20** interacting with the 1M17 protein. The amino acid residues of EGFR which had interactions with compound **H20** were labeled. In the proposed binding mode, compound **H20** was nicely bound to the ATP binding pocket of EGFR through hydrogen bond interactions and hydrophobic interactions.

In the binding model, compound **H20** was well bound to the EGFR protein with Gln767, Thr766, Arg752, Val702, Leu694, Leu820, Ala719, the seven amino acids located in the binding pocket of the protein, playing an important role in the combination with compound **H20**. As we can see from Figure 4 and Figure 5, Gln767 formed conventional hydrogen bonds and Thr766 formed Van der Waals bonds with the nitrogen atom of the Schiff base group, which enhanced the combination activity of compound **H20**. Moreover, a carbon–hydrogen bond and π-sulfur bonds were found between Arg828 and Cys751 with a benzene ring. Meanwhile the Leu694 formed three alkyl bonds with the methyl, benzene ring and dihydropyrazole ring. Alkyl and π-alkyl bonds were displayed in presence of Leu820 and dihydropyrazole ring and benzene ring, respectively. Moreover, Ala719 also formed π-alkyl bonds with the benzene ring. Naphthalene was also bonded with Val702 by a π-alkyl bond. In molecular docking 3D modeling, compound **H20** was nicely bound to the ATP binding site through the hydrogen bond with the backbone of Gln767 (distance = 2.4 Å) which increased the binding affinity dramatically in theory, as displayed in Figure 5.

**Figure 6:**
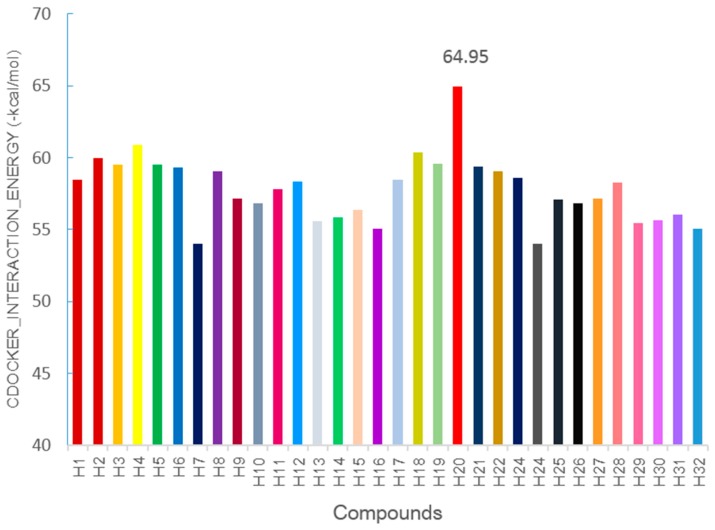
The histogram about CDOCKER_INTERACTION_ENERGY (−kcal/mol) of compounds (**H1**–**H32**) for EGFR.

In addition, the predicted binding interaction energy was used as the criterion for ranking; the estimated interaction energies of other synthesized compounds ranged from −64.95 to −52.99 kcal/mol, as displayed in Figure 6 with a histogram. The selected compound of **H20** had a best estimated binding free energy of −64.95 kcal/mol for EGFR. These molecular docking results, along with the biological assay data, suggested that compound **H20** is a potential inhibitor of EGFR.

## 3. Experimental Section

### 3.1. General Information

All chemicals and reagents used in current study were commercially available analytical or chemically pure grade reagents, unless otherwise indicated. All the reactions were monitored by thin-layer chromatography (TLC) on glass-backed silica gel sheets (silica GF 254) and visualized using UV (254 or 365 nm). Column chromatography separations were performed on silica gel (200–300 mesh) using EtOAc/petroleum ether as eluents. Melting points (uncorrected) were determined using an X-4 MP apparatus (Taike Corp, Beijing, China). All the ^1^H-NMR and ^13^C-NMR spectra were recorded using a model DPX400 spectrometer (Bruker, Billerica, MA, USA) in DMSO-*d*_6_ and chemical shifts were reported in δ (ppm) units relative to the internal standard tetramethylsilane (TMS). Mass spectra (MS) were recorded using a Mariner System 5304 mass spectrometer (Isoprime, Manchester, UK). 

### 3.2. General Synthesis Procedure of Benzohydrazide Derivatives Containing Dihydropyrazole Moieties

#### 3.2.1. Synthesis of Methyl 4-Hydrazinylbenzoate (**B**)

4-Hydrazinylbenzoic acid (3.0 g, 0.020 mol) was suspended in anhydrous methanol (30 mL) and thionyl chloride (5 mL) was added dropwise at 0 °C over 30 min. Then the reaction mixture was stirred at room temperature for 20–24 h, the precipitate that formed was filtered, washed with petroleum ether and ethanol (5:1) three times to remove residual 4-hydrazinylbenzoic acid, and dried to give the title 4-hydrazinylbenzoate.

#### 3.2.2. General Synthetic Procedure for Diverse Substituted Chalcones **E1**–**E16**

The diverse substituted chalcones **E1**–**E16** were synthesized by reacting the appropriate substituted acetophenone (3.0 mmol) and one equivalent of naphthaldehyde (0.5 g, 3.0 mmol) using 40% potassium hydroxide (2 mL) as catalyst in ethanol (20 mL). The reaction mixture was stirred at room temperature for 6–8 h, the yellow precipitate that formed was filtered, washed with petroleum ether and ethanol (5:1) three times to remove impurities, and dried to give the diverse substituted chalcones.

#### 3.2.3. General Synthetic Procedure for Diverse Substituted Chalcones **F1**–**F16**

Added acetic acid (1 mL) was used as an catalytic agent. The cyclization reaction of different substituted chalcones **E1**–**E16** (3.0 mmol) and methyl 4-hydrazinylbenzoate (3.0 mmol) was performed in refluxing ethanol (20 mL) at 80 °C for 20–24 h to afford compounds **F1**–**F16**, as monitored by thin layer chromatography (TLC). The crude products were purified in good yields by recrystallization from ethanol, ethyl acetate and acetone (1:1:0.05).

#### 3.2.4. General Synthetic Procedure for Compounds **G1**–**G16**

Compounds **F1**–**F16** (3.0 mmol) were dissolved in DMF (10 mL) and excess hydrazine hydrate (80%, 5 mL) was added dropwise in methanol (20 mL). The solution was refluxed at 70 °C for 20–24 h, and then poured into water (50 mL) and extracted with ethyl acetate (3 × 30 mL). The organic layer was collected, and washed with brine. The organic layer was dried over anhydrous sodium sulfate and concentrated in vacuo to obtain yields 60%–80% of crude products **G1**–**G16**.

#### 3.2.5. General Synthetic Procedure for the Title Compounds **H1**–**H32**

Equimolar amounts of compounds **G1**–**G16** (2.0 mmol) and 3- or 4-methoxybenzaldehyde (2.0 mmol) were dissolved in ethanol (20 mL), then acetic acid (1 mL) was added as catalytic agent. The reaction mixture solution continued to stirred at room temperature for 6–8 h. The resulting solid was extracted with petroleum ether for column chromatography. Column chromatography was performed using silica gel (200–300 mesh), eluting with ethyl acetate and petroleum ether (1:2, *v*/*v*), to give the title products. All of the target compounds gave satisfactory analytical and ^1^H-NMR (in Supplementary Materials) and MS spectroscopic data, in accordance with their depicted structures.

*(E)-N′-(3-Methoxybenzylidene)-4-(5-(naphthalen-2-yl)-3-phenyl-4,5-dihydro-1H-pyrazol-1-yl)benzo-hydrazide* (**H1**). Yellow powder. Yield: 75.01%. m.p. 238–240 °C. ^1^H-NMR (DMSO-*d*_6_): 3.28–3.34 (dd, *J*_1_ = 5.44 Hz, *J*_2_ = 17.72 Hz, 1H); 3.79 (s, 3H); 4.04–4.11 (dd, *J*_1_ = 12.32 Hz, *J*_2_ = 17.56 Hz, 1H); 5.81–5.85 (dd, *J*_1_ = 5.40 Hz, *J*_2_ = 12.12 Hz, 1H); 6.97–6.99 (d, *J* = 7.96 Hz, 1H); 7.13–7.15 (d, *J* = 8.88 Hz, 2H); 7.23 (s, 1H); 7.32–7.36 (m, 1H); 7.38–7.53 (m, 6H); 7.73–7.75 (d, *J* = 8.80 Hz, 2H); 7.83–7.84 (m, 2H); 7.88–7.93 (m, 4H); 8.33 (s, 1H); 11.55 (s, 1H). ^13^C-NMR (DMSO-*d*_6_): 163.07, 161.08, 149.84, 139.88, 133.40, 132.34, 129.71, 129.66, 129.40, 129.21, 128.13, 128.08, 127.57, 126.97, 126.50, 125.07, 124.26, 123.28, 114.75, 112.49, 63.20, 60.40, 55.72, 43.22. MS *m/z*: [M + H]^+^ 525.6 (C_34_H_28_N_4_O_2_). Anal. Calcd. for C_34_H_28_N_4_O_2_: C, 78.34; H, 5.45; N, 10.38. Found: C, 77.84; H, 5.38; N, 10.68.

*(E)-N′-(3-Methoxybenzylidene)-4-(5-(naphthalen-2-yl)-3-(o-tolyl)-4,5-dihydro-1H-pyrazol-1-yl)benzo-hydrazide* (**H2**).Yellow powder. Yield: 84.15%. m.p. 242–244 °C. ^1^H-NMR (DMSO-*d*_6_): 2.76 (s,3H); 3.34–3.39 (dd, *J*_1_ = 5.48 Hz, *J*_2_ = 11.92 Hz, 1H); 3.78 (s, 3H); 4.11–4.18 (dd, *J*_1_ = 12.12 Hz, *J*_2_ = 17.84 Hz, 1H); 5.74–5.79 (dd, *J*_1_ = 5.56 Hz, *J*_2_ = 12.36 Hz, 1H); 6.97–6.99 (d, *J* = 7.72 Hz, 1H); 7.09–7.11 (d, *J* = 8.80 Hz, 2H); 7.23–7.41 (m, 7H); 7.48–7.53 (m, 3H); 7.73–7.75 (d, *J* = 8.72 Hz, 2H); 7.87–7.92 (m, 4H); 8.32 (s, 1H); 11.54 (s, 1H). ^13^C-NMR (DMSO-*d*_6_): 163.12, 161.08, 148.84, 139.90, 133.46, 132.34, 129.71, 129.66, 129.50, 129.21, 128.13, 127.57, 126.97, 126.50, 125.07, 124.26, 123.28, 114.75, 112.49, 60.33, 55.73, 43.42. MS *m/z*: [M + H]^+^ 539.6 (C_35_H_30_N_4_O_2_). Anal. Calcd. for C_35_H_30_N_4_O_2_: C, 78.04; H, 5.61; N, 10.40. Found: C, 77.92; H, 5.56; N, 10.34.

*(E)-N′-(3-Methoxybenzylidene)-4-(5-(naphthalen-2-yl)-3-(m-tolyl)-4,5-dihydro-1H-pyrazol-1-yl)benzo-hydrazide* (**H3**). Yellow powder. Yield: 87.67%. m.p. 232–234 °C. ^1^H-NMR (DMSO-*d*_6_): 2.37 (s, 3H); 3.26–3.32 (dd, *J*_1_ = 5.12 Hz, *J*_2_ = 17.64 Hz, 1H); 3.79 (s, 3H); 4.01–4.09 (dd, *J*_1_ = 12.16 Hz, *J*_2_ = 17.64 Hz, 1H); 4.37–4.40 (t, 1H); 5.80–5.84 (dd, *J*_1_ = 5.36 Hz, *J*_2_ = 12.16 Hz, 1H); 6.97–6.99 (d, *J* = 8.08 Hz, 1H); 7.13–7.15 (d, *J* = 8.68 Hz, 2H); 7.23–7.24 (d, *J* = 6.12 Hz, 2H); 7.33–7.39 (m, 3H); 7.49–7.51 (m, 2H); 7.60–7.62 (d, *J* = 7.72 Hz, 1H); 7.68 (s, 1H); 7.73–7.76 (d, *J* = 8.60 Hz, 2H); 7.86–7.93 (m, 4H); 8.33 (s, 1H); 11.55 (s, 1H). ^13^C-NMR (DMSO-*d*_6_): 163.07, 161.07, 149.84, 139.88, 133.40, 132.34, 129.71, 129.66, 129.40, 129.21, 128.13, 128.08, 127.57, 126.97, 126.50, 125.07, 124.26, 123.28, 114.75, 112.49, 60.23, 55.72, 43.35. MS *m/z*: [M + H]^+^ 539.6 (C_35_H_30_N_4_O_2_). Anal. Calcd. for C_35_H_30_N_4_O_2_: C, 78.04; H, 5.61; N, 10.40. Found: C, 77.94; H, 5.54; N, 10.35.

*(E)-N′-(3-Methoxybenzylidene)-4-(5-(naphthalen-2-yl)-3-(p-tolyl)-4,5-dihydro-1H-pyrazol-1-yl)benzo-hydrazide* (**H4**). Yellow powder. Yield: 87.18%. m.p. 233–235 °C. ^1^H-NMR (DMSO-*d*_6_): 2.35 (s, 3H); 3.25–3.30 (dd, *J*_1_ = 4.24 Hz, *J*_2_ = 17.60 Hz, 1H); 3.79 (s, 3H); 4.02–4.08 (dd, *J*_1_ = 12.52 Hz, *J*_2_ = 17.80 Hz, 1H); 5.78–5.82 (dd, *J*_1_ = 5.52 Hz, *J*_2_ = 12.24 Hz, 1H); 6.97–6.99 (d, *J* = 8.04 Hz, 1H); 7.11–7.13 (d, *J* = 8.80 Hz, 2H); 7.23–7.29 (m, 4H); 7.32–7.40 (m, 2H); 7.49–7.51 (m, 2H); 7.71–7.75 (m, *J*_1_ = 4.12 Hz, *J*_2_ = 4.88 Hz, 4H); 7.87–7.93 (m, 4H); 8.33 (s, 1H); 11.54 (s, 1H). ^13^C-NMR (DMSO-*d*_6_): 163.09, 161.05, 150.14, 138.88, 133.40, 129.71, 129.66, 129.40, 129.21, 128.13, 128.08, 127.57, 126.97, 126.50, 125.07, 124.26, 123.28, 114.75, 112.49, 60.54, 55.72, 43.35. ESI- MS *m/z*: [M + H]^+^ 539.6 (C_35_H_30_N_4_O_2_). Anal. Calcd. for C_35_H_30_N_4_O_2_: C, 78.04; H, 5.61; N, 10.40. Found: C, 77.95; H, 5.56; N, 10.36.

*(E)-4-(3-(4-Ethylphenyl)-5-(naphthalen-2-yl)-4,5-dihydro-1H-pyrazol-1-yl)-N′-(3-methoxybenzylidene)-benzohydrazide* (**H5**). Yellow powder. Yield: 76.02%. m.p. 241–243 °C. ^1^H-NMR (DMSO-*d*_6_): 3.25–3.30 (dd, *J*_1_ = 5.20 Hz, *J*_2_ = 17.64 Hz, 1H); 3.79–3.81 (d, *J* = 9.12 Hz, 6H); 4.00–4.08 (dd, *J*_1_ = 12.04 Hz, *J*_2_ = 17.52 Hz, 1H); 5.76–5.80 (dd, *J*_1_ = 5.16 Hz, *J*_2_ = 12.08 Hz, 1H); 6.97–7.04 (m, 3H); 7.09–7.11 (d, *J* = 8.76 Hz, 2H); 7.23 (s, 2H); 7.30–7.40 (m, 3H); 7.49–7.51 (m, 2H); 7.71–7.78 (m, 4H); 7.87–7.93 (m, 4H); 8.32 (s, 1H); 11.53 (s, 1H). ^13^C-NMR (DMSO-*d*_6_): 163.07, 161.06, 160.56, 149.95, 149.85, 146.85, 146.77, 145.72, 140.01, 139.94, 133.40, 132.88, 129.62, 129.39, 128.92, 128.61, 128.23, 128.07, 127.59, 126.95, 126.63, 126.57, 125.02, 124.91, 124.27, 122.85, 114.75, 114.67, 112.26, 60.22, 55.72, 43.57, 43.44. MS *m/z*: [M + H]^+^ 553.2 (C_36_H_32_N_4_O_2_). Anal. Calcd. for C_36_H_32_N_4_O_2_: C, 78.24; H, 5.84; N, 10.14. Found: C, 78.12; H, 5.79; N, 10.11.

*(E)-N′-(3-Methoxybenzylidene)-4-(3-(4-methoxyphenyl)-5-(naphthalen-2-yl)-4,5-dihydro-1H-pyrazol-1-yl)-benzohydrazide* (**H6**). Yellow powder. Yield: 63.64%. m.p. 234–236 °C. ^1^H-NMR (DMSO-*d*_6_): 2.62–2.67 (dd, *J*_1_ = 7.52 Hz, *J*_2_ = 15.08 Hz, 2H); 3.25–3.31 (dd, *J*_1_ = 5.12Hz, *J*_2_ = 17.56 Hz, 1H); 3.79–3.84 (t, 3H); 4.00–4.08 (dd, *J*_1_ = 12.08 Hz, *J*_2_ = 17.92 Hz, 1H); 5.77–5.82 (dd, *J*_1_ = 5.36 Hz, *J*_2_ = 12.16 Hz, 1H); 6.97–6.99 (d, *J* = 7.84 Hz, 1H); 7.10–7.14 (m, 2H); 7.23 (s, 2H); 7.29–7.40 (m, 4H); 7.49–7.51 (m, 2H); 7.73–7.75 (d, *J* = 8.08 Hz, 4H); 7.87–7.92 (m, 5H); 8.33 (s, 1H); 11.54 (s, 1H). ^13^C-NMR (DMSO-d_6_): 163.07, 161.06, 149.96, 146.81, 146.72, 145.73, 139.95, 133.40, 132.88, 129.86, 129.64, 129.38, 128.92, 128.62, 128.24, 128.07, 127.58, 126.96, 126.64, 125.04, 124.26, 123.09, 114.75, 112.38, 63.08, 55.72, 43.57, 43.42. MS *m/z*: [M + H]^+^ 555.6 (C_35_H_30_N_4_O_3_). Anal. Calcd. for C_35_H_30_N_4_O_3_: C, 75.79; H, 5.45; N, 10.10. Found: C, 75.67; H, 5.43; N, 10.02.

*(E)-N′-(3-Methoxybenzylidene)-4-(5-(naphthalen-2-yl)-3-(3,4,5-trimethoxyphenyl)-4,5-dihydro-1H-pyrazol-1-yl)benzohydrazide* (**H7**). Yellow powder. Yield: 77.42%. m.p. 230–232 °C. ^1^H-NMR (DMSO-*d*_6_): 3.35–3.41 (dd, *J*_1_ = 5.68 Hz, *J*_2_ = 17.56 Hz, 1H); 3.70 (s, 3H); 3.79 (s, 3H); 3.86 (s, 6H); 4.01–4.05 (dd, *J*_1_ = 6.28 Hz, *J*_2_ = 15.16 Hz, 1H); 5.82–5.86 (dd, *J*_1_ = 5.12 Hz, *J*_2_ = 16.40 Hz, 1H); 6.97–6.99 (d, *J* = 8.04 Hz, 1H); 7.10–7.11 (m, 4H); 7.23 (s, 2H); 7.33–7.40 (m, 2H); 7.49–7.51 (m, 2H); 7.72–7.74 (d, *J* = 8.68 Hz, 2H); 7.85–7.94 (m, 4H); 8.33 (s, 1H); 11.53 (s, 1H). ^13^C-NMR (DMSO-*d*_6_): 163.92, 163.63, 162.97, 162.32, 162.23, 161.09, 160.70, 147.83, 146.98, 146.16, 139.53, 136.01, 135.94, 135.87, 133.37, 132.92, 129.70, 129.64, 129.37, 128.95, 128.08, 127.53, 127.13, 126.67, 125.14, 124.24, 123.98, 114.75, 112.91, 109.54, 109.36, 104.85, 104.68, 104.51, 63.60, 55.72, 42.98. MS *m/z*: [M + H]^+^ 615.7 (C_37_H_34_N_4_O_5_). Anal. Calcd. for C_37_H_34_N_4_O_5_: C, 72.30; H, 5.58; N, 9.11. Found: C, 72.18; H, 5.56; N, 9.08.

*(E)-4-(3-(4-Ethoxyphenyl)-5-(naphthalen-2-yl)-4,5-dihydro-1H-pyrazol-1-yl)-N′-(3-methoxybenzylidene)-benzohydrazide* (**H8**). Yellow powder. Yield: 82.31%. m.p. 233–235 °C. ^1^H-NMR (DMSO-*d*_6_): 1.32–1.36 (t, 3H); 3.23–3.29 (dd, *J*_1_ = 5.12 Hz, *J*_2_ = 17.56 Hz, 1H); 3.78 (s, 3H); 3.99–4.10 (m, 3H); 5.74–5.78 (dd, *J*_1_ = 5.52 Hz, *J*_2_ = 12.08 Hz, 1H); 6.96–7.02 (dd, *J*_1_ = 1.76 Hz, *J*_2_ = 11.40 Hz, 3H); 7.09–7.11 (d, *J* = 8.84 Hz, 2H); 7.23 (s, 2H); 7.32–7.40 (m, 2H); 7.47–7.52 (m, 2H); 7.73–7.77 (m, 4H); 7.86–7.92 (m, 4H); 8.33 (s, 1H); 11.53 (s, 1H). ^13^C-NMR (DMSO-*d*_6_): 163.01, 161.07, 159.98, 149.86, 146.85, 146.76, 140.01, 133.40, 132.87, 129.62, 129.38, 128.91, 128.43, 128.17, 128.07, 127.59, 126.95, 126.56, 125.02, 124.75, 124.27, 122.82, 115.08, 112.24, 63.69, 63.24, 55.72, 43.45. MS *m/z*: [M + H]^+^ 569.6 (C_36_H_32_N_4_O_3_). Anal. Calcd. for C_36_H_32_N_4_O_3_: C, 76.04; H, 5.67; N, 9.85. Found: C, 75.90; H, 5.65; N, 9.81.

*(E)-4-(3-(2-Fluorophenyl)-5-(naphthalen-2-yl)-4,5-dihydro-1H-pyrazol-1-yl)-N′-(3-methoxybenzylidene)-benzohydrazide* (**H9**). Yellow powder. Yield: 83.1%. m.p. 235–237 °C. ^1^H-NMR (DMSO-*d*_6_): 3.31–3.36 (dd, *J*_1_ = 5.52 Hz, *J*_2_ = 17.64 Hz, 1H); 3.79 (s, 3H); 4.11–4.19 (dd, *J*_1_ = 12.52 Hz, *J*_2_ = 18.84 Hz, 1H); 5.80–5.85 (dd, *J*_1_ = 5.12 Hz, *J*_2_ = 12.16 Hz, 1H); 6.97–6.99 (d, *J* = 7.68 Hz, 1H); 7.14–7.16 (d, *J* = 8.48 Hz, 2H); 7.23 (s, 2H); 7.29–7.33 (m, 3H); 7.40–7.52 (m, 4H); 7.74–7.76 (d, *J* = 8.44 Hz, 2H); 7.88–8.02 (m, 5H); 8.33 (s, 1H); 11.55 (s, 1H). ^13^C-NMR (DMSO-*d*_6_): 163.97, 163.08, 161.91, 161.07, 149.52, 146.87, 146.76, 139.32, 133.39, 132.90, 129.67, 129.40, 128.98, 128.97, 128.94, 128.75, 128.24, 128.07, 127.57, 126.61, 125.08, 124.24, 123.30, 116.16, 114.75, 112.49, 63.42, 60.43, 55.72, 43.42. MS *m/z*: [M + H]^+^ 543.6 (C_34_H_27_FN_4_O_2_). Anal. Calcd. for C_34_H_27_FN_4_O_2_: C, 75.26; H, 5.02; F, 3.50; N, 10.33. Found: C, 75.16; H, 5.01; F, 3.48; N, 10.30.

*(E)-4-(3-(3-Fluorophenyl)-5-(naphthalen-2-yl)-4,5-dihydro-1H-pyrazol-1-yl)-N′-(3-methoxybenzylidene)-benzohydrazide* (**H10**). Yellow powder. Yield: 74.96%. m.p. 241–243 °C.^1^H-NMR (DMSO-*d*_6_): 3.30–3.36 (dd, *J*_1_ = 5.28 Hz, *J*_2_ = 17.72 Hz, 1H); 3.79 (s, 3H); 4.02–4.10 (dd, *J*_1_ = 12.96 Hz, *J*_2_ = 18.32 Hz, 1H); 5.84–5.89 (dd, *J*_1_ = 5.36 Hz, *J*_2_ = 12.28 Hz, 1H); 6.97–6.99 (d, *J* = 7.68 Hz, 1H); 7.15–7.17 (d, *J* = 8.76 Hz, 2H); 7.24–7.28 (m, 3H); 7.33–7.40 (m, 2H); 7.49–7.51 (m, 3H); 7.63–7.67 (m, 2H); 7.73–7.76 (d, *J* = 8.72 Hz, 2H); 7.88–7.93 (m, 4H); 8.33 (s, 1H); 11.56 (s, 1H). ^13^C-NMR (DMSO-*d*_6_): 163.87, 163.08, 162.31, 161.07, 149.32, 146.87, 146.66, 139.82, 133.39, 132.90, 129.66, 129.40, 128.99, 128.97, 128.94, 128.77, 128.71, 128.24, 128.07, 127.57, 126.61, 125.08, 124.24, 123.30, 116.16, 114.74, 112.49, 63.42, 60.23, 55.72, 43.42.MS *m/z*: [M + H]^+^ 543.6 (C_34_H_27_FN_4_O_2_). Anal. Calcd. for C_34_H_27_FN_4_O_2_: C, 75.26; H, 5.02; N, 10.33. Found: C, 75.14; H, 5.00; N, 10.31.

*(E)-4-(3-(4-Fluorophenyl)-5-(naphthalen-2-yl)-4,5-dihydro-1H-pyrazol-1-yl)-N′-(3-methoxybenzylidene)-benzohydrazide* (**H11**). Yellow powder. Yield: 80.56%. m.p. 235–237 °C. ^1^H-NMR (DMSO-*d*_6_): 3.28–3.32 (dd, *J*_1_ = 5.16 Hz, *J*_2_ = 17.84 Hz, 1H); 3.79 (s, 3H); 4.03–4.10 (dd, *J*_1_ = 10.04 Hz, *J*_2_ = 17.64 Hz, 1H); 5.81–5.85 (dd, *J*_1_ = 6.40 Hz, *J*_2_ = 12.28 Hz, 1H); 6.97–6.99 (d, *J* = 7.68 Hz, 1H); 7.12–7.14 (d, *J* = 8.80 Hz, 2H); 7.23 (s, 2H); 7.29–7.34 (m, 3H); 7.38–7.40 (d, *J* = 9.88 Hz, 1H); 7.50–7.53 (m, 2H); 7.72–7.75 (d, *J* = 8.72 Hz, 2H); 7.86–7.93 (m, 6H); 8.32 (s, 1H); 11.54 (s, 1H). ^13^C-NMR (DMSO-*d*_6_): 163.87, 162.35, 161.07, 150.32, 146.87, 146.66, 139.82, 134.39, 132.90, 129.66, 129.40, 128.99, 128.97 128.94, 128.77, 128.71, 128.24, 128.07, 127.57, 126.61, 125.08, 124.24, 123.30, 116.16, 114.74, 112.49, 63.45, 60.33, 55.74, 43.45. MS *m/z*: [M + H]^+^ 543.6 (C_34_H_27_FN_4_O_2_). Anal. Calcd. for C_34_H_27_FN_4_O_2_: C, 75.26; H, 5.02; N, 10.33. Found: C, 75.15; H, 4.99; N, 10.29.

*(E)-4-(3-(3,5-Difluorophenyl)-5-(naphthalen-2-yl)-4,5-dihydro-1H-pyrazol-1-yl)-N′-(3-methoxybenzylidene) benzohydrazide* (**H12**). Yellow powder. Yield: 77.14%. m.p. 225–227 °C. ^1^H-NMR (DMSO-*d*_6_): 3.30–3.36 (dd, *J*_1_ = 5.44 Hz, *J*_2_ = 18.08 Hz, 1H); 3.79 (s, 3H); 4.00–4.07 (dd, *J*_1_ = 12.40 Hz, *J*_2_ = 17.80 Hz, 1H); 5.88–5.92 (dd, *J*_1_ = 5.60 Hz, *J*_2_ = 12.40 Hz, 1H); 6.97–6.99 (d, *J* = 8.04 Hz, 1H); 7.17–7.23 (m, 4H); 7.28–7.40 (m, 3H); 7.48–7.54 (m, 4H); 7.73–7.76 (d, *J* = 8.78 Hz, 2H); 7.88–7.94 (m, 4H); 8.33 (s, 1H); 11.57 (s, 1H). ^13^C-NMR (DMSO-*d*_6_): 163.08, 161.01, 153.43, 149.96, 146.85, 146.58, 133.93, 139.14, 133.41, 132.89, 129.66, 129.63, 129.37, 128.82, 128.22, 128.07, 127.86, 127.56, 126.98, 126.59, 124.92, 124.26, 123.18, 114.75, 112.44, 104.02, 63.15, 56.46, 55.72, 43.52. MS *m/z*: [M + H]^+^ 561.6 (C_34_H_26_F_2_N_4_O_2_). Anal. Calcd. for C_34_H_26_F_2_N_4_O_2_: C, 72.85; H, 4.67; N, 9.99. Found: C, 72.74; H, 4.66; N, 9.96.

*(E)-4-(3-(3-Chlorophenyl)-5-(naphthalen-2-yl)-4,5-dihydro-1H-pyrazol-1-yl)-N′-(3-methoxybenzylidene)-benzohydrazide* (**H13**). Yellow powder. Yield: 87.17%. m.p. 231–233 °C. ^1^H-NMR (DMSO-*d*_6_): 3.30–3.36 (dd, *J*_1_ = 5.44 Hz, *J*_2_ = 17.76 Hz, 1H); 3.79 (s, 3H); 4.01–4.08 (dd, *J*_1_ = 12.16 Hz, *J*_2_ = 15.84 Hz, 1H); 5.84–5.88 (dd, *J*_1_ = 5.52 Hz, *J*_2_ = 12.36 Hz, 1H); 6.97–6.99 (d, *J* = 8.08 Hz, 1H); 7.15–7.17 (d, *J* = 8.84 Hz, 2H); 7.23 (s, 2H); 7.33–7.44 (m, 3H); 7.49–7.51 (m, 2H) 7.60–7.62 (d, *J* = 8.84 Hz, 1H); 7.74–7.76 (d, *J* = 8.76 Hz, 2H); 7.80–7.82 (d, *J* = 7.88 Hz, 1H); 7.87–7.93 (m, 4H); 8.01 (s, 1H); 8.33 (s, 1H); 11.57 (s, 1H). ^13^C-NMR (DMSO-*d*_6_): 170.09, 163.07, 161.08, 149.72, 146.89, 146.43, 139.54, 134.12, 133.39, 132.90, 131.37, 129.67, 129.40, 129.25, 128.20, 128.08, 127.56, 126.98, 126.63, 125.11, 124.24, 123.51, 114.75, 112.60, 63.20, 60.32, 55.72, 43.42. MS *m/z*: [M + H]^+^ 560.0 (75%), [M + 2 + H]^+^ 562.0 (25%), (C_34_H_27_ClN_4_O_2_). Anal. Calcd. for C_34_H_27_ClN_4_O_2_: C, 73.05; H, 4.87; N, 10.02;. Found: C, 72.90; H, 4.85; N, 9.98.

*(E)-4-(3-(4-Chlorophenyl)-5-(naphthalen-2-yl)-4,5-dihydro-1H-pyrazol-1-yl)-N′-(3-methoxybenzylidene)-benzohydrazide* (**H14**). Yellow powder. Yield: 72.41%. m.p. 236–238 °C. ^1^H-NMR (DMSO-*d*_6_): 3.32–3.38 (dd, *J*_1_ = 5.40 Hz, *J*_2_ = 17.60 Hz, 1H); 3.79 (s, 3H); 4.02–4.09 (dd, *J*_1_ = 11.84 Hz, *J*_2_ = 15.40 Hz, 1H); 5.82–5.87 (dd, *J*_1_ = 6.96 Hz, *J*_2_ = 12.44 Hz, 1H); 6.97–6.99 (d, *J* = 7.68 Hz, 1H); 7.13–7.15 (d, *J* = 8.80 Hz, 2H); 7.23 (s, 2H); 7.36–7.40 (m, 2H); 7.49–7.54 (m, 4H); 7.73–7.75 (d, *J* = 9.08 Hz, 2H); 7.83–7.93 (m, 6H); 8.33 (s, 1H); 11.58 (s, 1H). ^13^C-NMR (DMSO-*d*_6_): 170.11, 163.08, 161.07, 148.79, 146.89, 146.47, 139.53, 134.11, 133.39, 132.90, 131.27, 129.67, 129.40, 129.25, 128.20, 128.08, 127.56, 126.98, 126.63, 125.11, 124.24, 123.51, 114.75, 112.60, 63.20, 60.42, 55.72, 43.42. MS *m/z*: [M + H]^+^ 560.0 (75%), [M + 2 + H]^+^ 562.0 (25%), (C_34_H_27_ClN_4_O_2_). Anal. Calcd. for C_34_H_27_ClN_4_O_2_: C, 73.05; H, 4.87; N, 10.02. Found: C, 72.91; H, 4.86; N, 9.99.

*(E)-4-(3-(3,4-Dichlorophenyl)-5-(naphthalen-2-yl)-4,5-dihydro-1H-pyrazol-1-yl)-N′-(3-methoxy-benzylidene)benzohydrazide* (**H15**). Yellow powder. Yield: 71.11%. m.p. 234–236 °C. ^1^H-NMR (DMSO-*d*_6_): 3.32–3.37 (dd, *J*_1_ = 5.44 Hz, *J*_2_ = 16.48 Hz, 1H); 3.79 (s, 3H); 4.01–4.09 (dd, *J*_1_ = 12.36 Hz, *J*_2_ = 17.72 Hz, 1H); 5.86–5.90 (dd, *J*_1_ = 5.72 Hz, *J*_2_ = 12.32 Hz, 1H); 6.97–6.99 (d, *J* = 7.96 Hz, 1H); 7.16–7.18 (d, *J* = 8.80 Hz, 2H); 7.23 (s, 2H); 7.33–7.40 (m, 2H); 7.50–7.52 (m, 2H); 7.71–7.75 (m, 3H); 7.80–7.83 (m, 1H); 7.87–7.93 (m, 4H); 8.01–8.02 (m, 1H); 8.32 (s, 1H); 11.56 (s, 1H). ^13^C-NMR (DMSO-*d*_6_): 163.07, 161.08, 149.79, 146.78, 146.47, 139.52, 134.12, 133.39, 132.87, 131.27, 129.67, 129.40, 129.25, 128.20, 128.08, 127.56, 126.98, 126.63, 125.11, 124.24, 123.51, 114.75, 112.60, 60.52, 55.72, 43.42. MS *m/z*: [M + H]^+^ 594.5 (75%), [M + 2 + H]^+^ 598.5 (25%), (C_34_H_26_Cl_2_N_4_O_2_). Anal. Calcd. for C_34_H_26_Cl_2_N_4_O_2_: C, 68.81; H, 4.42; N, 9.44; O, 5.39. Found: C, 68.69; H, 4.41; N, 9.41.

*(E)-4-(3-(4-Bromophenyl)-5-(naphthalen-2-yl)-4,5-dihydro-1H-pyrazol-1-yl)-N′-(3-methoxybenzylidene)-benzohydrazide* (**H16**). Yellow powder. Yield: 77.83%. m.p. 245–247 °C. ^1^H-NMR (DMSO-*d*_6_): 3.27–3.33 (dd, *J*_1_ = 5.40 Hz, *J*_2_ = 17.60 Hz, 1H); 3.79 (s, 3H); 4.02–4.09 (dd, *J*_1_ = 12.28 Hz, *J*_2_ = 17.68 Hz, 1H); 5.82–5.87 (dd, *J*_1_ = 5.64 Hz, *J*_2_ = 12.28 Hz, 1H); 6.97–6.99 (d, *J* = 7.80 Hz, 1H); 7.13–7.15 (d, *J* = 8.76 Hz, 2H); 7.23 (s, 2H); 7.32–7.40 (m, 2H); 7.49–7.51 (m, 2H); 7.65–7.67 (d, *J* = 8.52 Hz, 2H); 7.72–7.78 (m, 4H); 7.87–7.93 (m, 4H); 8.32 (s, 1H); 11.55 (s,1H). ^13^C-NMR (DMSO-*d*_6_): 163.07, 161.08, 148.56, 146.82, 146.47, 139.71, 133.38, 132.90, 131.60, 129.67, 129.40, 128.94, 128.43, 128.25, 127.55, 126.98, 126.63, 125.11, 124.23, 123.53, 122.83, 114.75, 112.61, 63.40, 55.73, 43.12. MS *m/z*: [M + H]^+^ 604.5 (50%), [M + 2 + H]^+^ 606.5 (50%), (C_34_H_27_BrN_4_O_2_). Anal. Calcd. for C_34_H_27_BrN_4_O_2_: C, 67.67; H, 4.51; N, 9.28. Found: C, 67.54; H, 4.50; N, 9.25.

*(E)-N′-(4-Methoxybenzylidene)-4-(5-(naphthalen-2-yl)-3-phenyl-4,5-dihydro-1H-pyrazol-1-yl)benzo-hydrazide* (**H17**)*.* Orange powder. Yield: 75.36%. m.p. 234–236 °C. ^1^H-NMR (DMSO-*d*_6_): 3.27–3.33 (dd, *J*_1_ = 4.20 Hz, *J*_2_ = 17.68 Hz, 1H); 3.79 (s, 3H); 4.04–4.11 (dd, *J*_1_ = 10.44 Hz, *J*_2_ = 17.56 Hz, 1H); 5.80–5.85 (dd, *J*_1_ = 5.56 Hz, *J*_2_ = 12.24 Hz, 1H); 6.98–7.00 (d, *J* = 8.44 Hz, 2H); 7.12–7.14 (d, *J* = 8.80 Hz, 2H); 7.39–7.53 (m, 6H); 7.61–7.63 (d, *J* = 8.20 Hz, 2H); 7.72–7.74 (d, *J* = 8.68 Hz, 2H); 7.82–7.84 (d, *J* = 7.04 Hz, 2H); 7.88–7.93 (m, 4H); 8.29 (s,1H); 11.41 (s, 1H). ^13^C-NMR (DMSO-*d*_6_): 163.03, 161.01, 149.84, 139.88, 133.40, 132.34, 129.71, 129.66, 129.40, 129.21, 128.13, 128.08, 127.57, 126.97, 126.50, 125.07, 124.26, 123.28, 114.75, 112.49, 63.20, 60.23, 55.73, 43.35. MS m/z: [M + H]^+^ 525.6 (C_34_H_28_N_4_O_2_). Anal. Calcd. for C_34_H_28_N_4_O_2_: C, 77.84; H, 5.38; N, 10.68. Found: C, 76.69; H, 5.39; N, 10.42.

*(E)-N′-(4-Methoxybenzylidene)-4-(5-(naphthalen-2-yl)-3-(o-tolyl)-4,5-dihydro-1H-pyrazol-1-yl)benzo-hydrazide* (**H18**). Yellow powder. Yield: 70.12%. m.p. 240–242 °C. ^1^H-NMR (DMSO-*d*_6_): 2.76 (s, 3H); 3.33–3.39 (dd, *J*_1_ = 5.44 Hz, *J*_2_ = 17.28 Hz, 1H); 3.79 (s, 3H); 4.10–4.18 (dd, *J*_1_ = 12.08 Hz, *J*_2_ = 17.44 Hz, 1H); 5.74–5.80 (dd, *J*_1_ = 5.44 Hz, *J*_2_ = 12.16 Hz, 1H); 6.98–7.00 (d, *J* = 8.40 Hz, 2H); 7.08–7.10 (d, *J* = 8.76 Hz, 2H); 7.25–7.32 (m, 2H); 7.36–7.41 (m, 2H); 7.48–7.53 (m, 3H); 7.61–7.63 (d, *J* = 8.12 Hz, 2H); 7.72–7.74 (d, *J* = 8.68 Hz, 2H); 7.87–7.93 (m, 4H); 8.29 (s, 1H); 11.39 (s, 1H). ^13^C-NMR (DMSO-*d*_6_): 163.08, 161.07, 149.88, 139.92, 133.45, 132.34, 129.73, 129.65, 129.41, 129.21, 128.13, 128.08, 127.57, 126.97, 126.50, 125.07, 124.26, 123.28, 114.75, 112.49, 63.22, 55.72, 43.42. MS *m/z*: [M + H]^+^ 539.6 (C_35_H_30_N_4_O_2_). Anal. Calcd. for C_35_H_30_N_4_O_2_: C, 78.04; H, 5.61; N, 10.40. Found: C, 77.91; H, 5.60; N, 10.36.

*(E)-N′-(4-Methoxybenzylidene)-4-(5-(naphthalen-2-yl)-3-(m-tolyl)-4,5-dihydro-1H-pyrazol-1-yl)benzo-hydrazide* (**H19**). Yellow powder. Yield: 82.68%. m.p. 230–232 °C. ^1^H-NMR (DMSO-*d*_6_): 2.37 (s, 3H); 3.26–3.32 (dd, *J*_1_ = 5.24 Hz, *J*_2_ = 17.56 Hz, 1H); 3.79 (s, 3H); 4.01–4.09 (dd, *J*_1_ = 12.08 Hz, *J*_2_ = 15.72 Hz, 1H); 5.80–5.84 (dd, *J*_1_ = 5.48 Hz, *J*_2_ = 12.32 Hz, 1H); 6.98–7.00 (d, *J* = 8.56 Hz, 2H); 7.12–7.14 (d, *J* = 8.84 Hz, 2H); 7.23–7.25 (d, *J* = 7.64 Hz, 1H); 7.33–7.40 (m, 2H); 7.49–7.51 (t, 2H); 7.60–7.63 (dd, *J*_1_ = 3.44 Hz, *J*_2_ = 4.32 Hz, 3H); 7.68 (s, 1H); 7.71–7.74 (d, *J* = 8.64 Hz, 2H); 7.86–7.93 (m, 4H); 8.29 (s, 1H); 11.40 (s, 1H). ^13^C-NMR (DMSO-*d*_6_): 163.03, 161.01, 149.84, 139.88, 133.40, 132.34, 129.71, 129.66, 129.40, 129.21, 128.13, 128.08, 127.57, 126.97, 126.50, 125.07, 124.26, 123.28, 114.75, 112.49, 63.20, 55.73, 43.35. MS *m/z*: [M + H]^+^ 539.6 (C_35_H_30_N_4_O_2_). Anal. Calcd. for C_35_H_30_N_4_O_2_: C, 78.04; H, 5.61; N, 10.40. Found: C, 77.66; H, 6.71; N, 9.46.

*(E)-N′-(4-Methoxybenzylidene)-4-(5-(naphthalen-2-yl)-3-(p-tolyl)-4,5-dihydro-1H-pyrazol-1-yl)benzo-hydrazide* (**H20**). Yellow powder. Yield: 73.91%. m.p. 241–243 °C. ^1^H-NMR (DMSO-*d*_6_): 2.35 (s, 3H); 3.23–3.29 (dd, *J*_1_ = 4.24 Hz, *J*_2_ = 17.64 Hz, 1H); 3.79 (s, 3H); 4.00–4.07 (dd, *J*_1_ = 12.20 Hz, *J*_2_ = 17.64 Hz, 1H); 5.76–5.81 (dd, *J*_1_ = 5.56 Hz, *J*_2_ = 12.16 Hz, 1H); 6.98–7.00 (d, *J* = 8.48 Hz, 2H); 7.10–7.13 (d, *J* = 8.72 Hz, 2H); 7.26–7.28 (d, *J* = 8.04 Hz, 2H); 7.37–7.40 (d, *J* = 8.40 Hz, 1H); 7.49–7.51 (m, 2H); 7.61–7.63 (d, *J* = 8.12 Hz, 2H); 7.71–7.73 (d, *J* = 8.04 Hz, 4H); 7.87–7.92 (m, 4H); 8.30 (s, 1H); 11.39 (s, 1H). ^13^C-NMR (DMSO-*d*_6_): 163.09, 161.05, 150.14, 138.58, 133.40, 129.71, 129.66, 129.40, 129.21, 128.13, 128.08, 127.57, 126.97, 126.50, 125.07, 124.26, 123.28, 114.75, 112.49, 60.54, 55.72, 43.35. MS *m/z*: [M + H]^+^ 539.6 (C_35_H_30_N_4_O_2_). Anal. Calcd. for C_35_H_30_N_4_O_2_: C, 78.04; H, 5.61; N, 10.40. Found: C, 77.60; H, 5.43; N, 10.36.

*(E)-4-(3-(4-Ethylphenyl)-5-(naphthalen-2-yl)-4,5-dihydro-1H-pyrazol-1-yl)-N′-(4-methoxybenzylidene)-benzohydrazide* (**H21**). Orange powder. Yield: 76.19%. m.p. 239–241 °C. ^1^H-NMR (DMSO-*d*_6_): 3.24–3.29 (dd, *J*_1_ = 4.40 Hz, *J*_2_ = 17.36 Hz, 2H); 3.79–3.81 (d, *J* = 2.84 Hz, 6H); 3.99–4.07 (dd, *J*_1_ = 11.36 Hz, *J*_2_ = 17.80 Hz, 1H); 5.74–5.78 (dd, *J*_1_ = 5.20 Hz, *J*_2_ = 11.60 Hz, 1H); 6.98–7.03 (m, 4H); 7.09–7.11 (d, *J* = 8.52 Hz, 2H); 7.38–7.40 (d, *J* = 8.52 Hz, 1H); 7.49–7.51 (m, 2H); 7.61–7.63 (d, *J* = 7.88 Hz, 2H); 7.71–7.78 (m, 4H); 7.86–7.93 (m, 5H); 8.29 (s, 1H); 11.40 (s, 1H). ^13^C-NMR (DMSO-*d*_6_): 163.07, 161.06, 160.70, 149.95, 149.85, 146.85, 146.77, 145.72, 140.01, 139.94, 133.40, 132.88, 129.62, 129.39, 128.92, 128.61, 128.23, 128.07, 127.59, 126.95, 126.63, 126.57, 125.02, 124.91, 124.27, 122.85, 114.75, 114.67, 112.26, 63.06, 55.76, 43.57, 43.44. MS *m/z*: [M + H]^+^ 553.2 (C_36_H_32_N_4_O_2_). Anal. Calcd. for C_36_H_32_N_4_O_2_: C, 78.24; H, 5.84; N, 10.14. Found: C, 74.84; H, 5.53; N, 9.85.

*(E)-N′-(4-Methoxybenzylidene)-4-(3-(4-methoxyphenyl)-5-(naphthalen-2-yl)-4,5-dihydro-1H-pyrazol-1-yl)-benzohydrazide* (**H22**). Yellow powder. Yield: 69.13%. m.p. 243–245 °C. ^1^H-NMR (DMSO-*d*_6_): 2.62–2.67 (dd, *J*_1_ = 7.56 Hz, *J*_2_ = 15.16 Hz, 2H); 3.25–3.30 (dd, *J*_1_ = 5.40 Hz, *J*_2_ = 7.64 Hz, 1H); 3.79–3.83 (t, 3H); 4.01–4.08 (dd, *J*_1_ = 12.36 Hz, *J*_2_ = 17.72 Hz, 1H); 5.77–5.82 (dd, *J*_1_ = 5.48 Hz, *J*_2_ = 12.12 Hz, 1H); 6.98–7.00 (d, *J* = 8.52 Hz, 2H); 7.10–7.13 (d, *J* = 8.80 Hz,2H); 7.29–7.31 (d, *J* = 8.20 Hz, 2H); 7.38–7.40 (d, *J* = 8.48 Hz, 1H); 7.49–7.51 (m, 2H); 7.61–7.63 (d, *J* = 8.28 Hz, 2H); 7.71–7.75 (m, 4H); 7.87–7.92 (m, 5H); 8.29 (s, 1H); 11.40 (s, 1H). ^13^C- NMR (DMSO-*d*_6_): 163.05, 161.06, 149.96, 146.81, 146.72, 145.73, 139.95, 133.40, 132.88, 129.86, 129.64, 129.38, 128.92, 128.62, 128.24, 128.07, 127.58, 126.96, 126.64, 125.04, 124.26, 123.09, 114.75, 112.38, 63.08, 55.77, 55.72, 43.57, 43.44. MS *m/z*: [M + H]^+^ 555.6 (C_35_H_30_N_4_O_3_). Anal. Calcd. for C_35_H_30_N_4_O_3_: C, 75.79; H, 5.45; N, 10.10. Found: C, 74.90; H, 5.99; N, 9.31.

*(E)-N′-(4-Methoxybenzylidene)-4-(5-(naphthalen-2-yl)-3-(3,4,5-trimethoxyphenyl)-4,5-dihydro-1H-pyrazol-1-yl)benzohydrazide* (**H23**). Yellow powder. Yield: 62.58%. m.p. 237–239 °C. ^1^H-NMR (DMSO-*d*_6_): 3.35–3.40 (dd, *J*_1_ = 5.64 Hz, *J*_2_ = 17.56 Hz, 1H); 3.70 (s,3H); 3.79 (s,3H); 3.86 (s, 6H); 4.01–4.08 (dd, *J*_1_ = 12.60 Hz, *J*_2_ = 17.56 Hz, 1H); 5.80–5.85 (dd, *J*_1_ = 5.20 Hz, *J*_2_ = 12.12 Hz, 1H); 6.98–7.00 (d, *J* = 8.60 Hz, 2H); 7.10–7.14 (m, 4H); 7.38–7.40 (d, *J* = 8.52 Hz, 1H); 7.49–7.51 (m, 2H); 7.61–7.63 (d, *J* = 8.20 Hz, 2H); 7.70–7.72 (d, *J* = 8.76 Hz, 2H); 7.85–7.94 (m, 4H); 8.29 (s, 1H); 11.39 (s, 1H). ^13^C-NMR (DMSO-*d*_6_): 163.95, 163.66, 162.97, 162.32, 162.23, 161.09, 160.70, 147.83, 146.98, 146.16, 139.53, 136.01, 135.94, 135.87, 133.37, 132.92, 129.70, 129.64, 129.37, 128.95, 128.08, 127.53, 127.00, 126.67, 125.14, 124.24, 123.98, 114.75, 112.91, 109.54, 109.36, 104.85, 104.68, 104.51, 63.65, 55.72, 42.98. MS *m/z*: [M + H]^+^ 615.7 (C_37_H_34_N_4_O_5_). Anal. Calcd. for C_37_H_34_N_4_O_5_: C, 72.30; H, 5.58; N, 9.11. Found: C, 71.41; H, 6.06; N, 8.31.

*(E)-4-(3-(4-Ethoxyphenyl)-5-(naphthalen-2-yl)-4,5-dihydro-1H-pyrazol-1-yl)-N′-(4-methoxybenzylidene)-benzohydrazide* (**H24**). Yellow powder. Yield: 76.15%. m.p. 237–239 °C. ^1^H-NMR (DMSO-*d*_6_): 1.32–1.36 (t, 3H); 3.23–3.29 (dd, *J*_1_ = 5.16 Hz, *J*_2_ = 17.60 Hz, 1H); 3.79 (s, 3H); 3.99–4.10 (m,3H); 5.74–5.79 (dd, *J*_1_ = 5.56 Hz, *J*_2_ = 12.12 Hz, 1H); 6.98–7.02 (m, 4H); 7.08–7.10 (d, *J* = 8.84 Hz, 2H); 7.37–7.40 (m, 1H); 7.49–7.51 (m, 2H); 7.61–7.63 (d, *J* = 8.24 Hz, 2H); 7.70–7.77 (m, 4H); 7.86–7.9 2(m, 4H); 8.29 (s ,1H); 11.39 (s, 1H). ^13^C- NMR (DMSO-*d*_6_): 163.07, 161.05, 159.98, 149.86, 146.85, 146.76, 140.01, 133.40, 132.87, 129.62, 129.38, 128.91, 128.23, 128.17, 128.07, 127.59, 126.95, 126.56, 125.02, 124.75, 124.27, 122.82, 115.08, 112.24, 63.69, 63.04, 55.72, 43.55. MS *m/z*: [M + H]^+^ 569.6 (C_36_H_32_N_4_O_3_). Anal. Calcd. for C_36_H_32_N_4_O_3_: C, 76.04; H, 5.67; N, 9.85. Found: C, 75.04; H, 5.71; N, 9.58.

*(E)-4-(3-(2-Fluorophenyl)-5-(naphthalen-2-yl)-4,5-dihydro-1H-pyrazol-1-yl)-N′-(4-methoxybenzylidene)-benzohydrazide* (**H25**). Yellow powder. Yield: 85.32%. m.p. 244–246 °C. ^1^H-NMR (DMSO-*d*_6_): 3.29–3.34 (dd, *J*_1_ = 5.52 Hz, *J*_2_ = 17.64 Hz, 1H); 3.79 (s, 3H); 4.11–4.18 (dd, *J_1_* = 12.20 Hz, *J_2_* = 17.68 Hz, 1H); 5.80–5.84 (dd, *J*_1_ = 5.60 Hz, *J*_2_ = 12.32 Hz, 1H); 6.99–7.01 (d, *J* = 8.16 Hz, 2H); 7.13–7.15 (d, *J* = 8.48 Hz, 2H); 7.28–7.33 (m, 2H); 7.40–7.52 (m, 5H); 7.61–7.63 (d, *J* = 7.92 Hz, 2H); 7.73–7.75 (d, *J* = 8.40 Hz,2H); 7.88–7.94 (m, 3H); 7.98–8.02 (m, 1H); 8.30 (s, 1H); 11.42 (s, 1H). ^13^C-NMR (DMSO-*d*_6_): 163.53, 162.31, 161.08, 149.22, 146.85, 146.76, 139.62, 133.42, 132.88, 129.66, 129.40, 128.99 128.97, 128.94, 128.77, 128.71, 128.24, 128.07, 127.57, 126.61, 125.08, 124.24, 123.30, 116.16, 114.74, 112.49, 63.23, 60.43, 55.73, 43.42. MS *m/z*: [M + H]^+^ 543.6 (C_34_H_27_FN_4_O_2_). Anal. Calcd. for C_34_H_27_FN_4_O_2_: C, 75.26; H, 5.02; N, 10.33. Found: C, 75.13; H, 5.01; N, 10.30.

*(E)-4-(3-(3-Fluorophenyl)-5-(naphthalen-2-yl)-4,5-dihydro-1H-pyrazol-1-yl)-N′-(4-methoxybenzylidene)-benzohydrazide* (**H26**). Yellow powder. Yield: 75.6%. m.p. 240–242 °C. ^1^H-NMR (DMSO-*d*_6_): 3.29–3.35 (dd, *J*_1_ = 5.56 Hz, *J*_2_ = 17.80 Hz, 1H); 3.79 (s, 3H); 4.02–4.09 (dd, *J*_1_ = 12.28 Hz, *J*_2_ = 17.72 Hz, 1H); 5.84–5.88 (dd, *J*_1_ = 5.64 Hz, *J*_2_ = 12.36 Hz, 1H); 6.99–7.01 (d, *J* = 8.48 Hz, 2H); 7.14–7.17 (d, *J* = 8.84 Hz, 2H); 7.24–7.28 (m, 1H); 7.38–7.40 (d, *J* = 8.52 Hz, 1H ); 7.48–7.54 (m, 3H); 7.61–7.67 (m, 4H); 7.72–7.74 (d, *J* = 8.68 Hz, 2H); 7.88–7.93 (m, 4H); 8.29 (s, 1H); 11.42 (s, 1H). ^13^C-NMR (DMSO-*d*_6_): 163.08, 162.32, 161.09, 149.11, 146.82, 146.64, 139.82, 133.32, 132.90, 129.65, 129.40, 128.99, 128.97, 128.95, 128.76, 128.72, 128.24, 128.07, 127.57, 126.65, 125.02, 124.24, 123.30, 116.16, 114.72, 112.45, 63.36, 60.24, 55.72, 43.42. MS *m/z*: [M + H]^+^ 543.6 (C_34_H_27_FN_4_O_2_). Anal. Calcd. for C_34_H_27_FN_4_O_2_: C, 75.26; H, 5.02; N, 10.33. Found: C, 75.14; H, 5.00; N, 10.31.

*(E)-4-(3-(4-Fluorophenyl)-5-(naphthalen-2-yl)-4,5-dihydro-1H-pyrazol-1-yl)-N'-(4-methoxybenzylidene)-benzohydrazide* (**H27**). Yellow powder. Yield: 62.50%. m.p. 236–238 °C. ^1^H-NMR (400 MHz, DMSO-*d*_6_): 3.28–3.34 (dd, *J*_1_ = 5.52 Hz, *J*_2_ = 17.72 Hz, 1H); 3.79 (s, 3H); 4.02–4.09 (dd, *J*_1_ = 7.08 Hz, *J*_2_ = 17.80 Hz,1H); 5.80–5.84 (dd, *J*_1_ = 5.68 Hz, *J*_2_ = 12.28 Hz, 1H); 6.99–7.01 (d, *J* = 8.48 Hz, 2H); 7.11–7.13 (d, *J* = 8.80 Hz, 2H); 7.29–7.33 (t,2H); 7.38–7.40 (m, 1H); 7.49–7.51 (m, 2H); 7.61–7.63 (d, *J* = 8.28 Hz, 2H); 7.72–7.74 (d, *J* = 8.68 Hz, 2H); 7.86–7.93 (m, 6H); 8.29 (s, 1H); 11.41 (s, 1H). ^13^C-NMR (100 MHz, DMSO-*d*_6_): 163.93, 163.06, 162.29, 161.07, 149.00, 146.87, 146.66, 139.82, 133.39, 132.90, 129.66, 129.40, 128.99, 128.97, 128.94, 128.94, 128.77, 128.71, 128.24, 128.07, 127.57, 126.61, 125.08, 124.24, 123.30, 116.16, 114.74, 112.49, 63.33, 60.23, 55.71, 43.42. MS *m/z*: [M + H]^+^ 543.6 (C_34_H_27_FN_4_O_2_). Anal. Calcd. for C_34_H_27_FN_4_O_2_: C, 75.26; H, 5.02; N, 10.33. Found: C, 73.87; H, 4.97; N, 10.06.

*(E)-4-(3-(3,5-Difluorophenyl)-5-(naphthalen-2-yl)-4,5-dihydro-1H-pyrazol-1-yl)-N′-(4-methoxy-benzylidene)benzohydrazide* (**H28**). Yellow powder. Yield: 69.3%. m.p. 238–240 °C. ^1^H-NMR (400 MHz, DMSO-*d*_6_): 3.30–3.36 (dd, *J*_1_ = 5.52 Hz, *J*_2_ = 17.88 Hz, 1H); 3.79 (s, 3H); 3.99–4.07 (dd, *J*_1_ = 12.36 Hz, *J*_2_ = 17.84 Hz, 1H); 5.87–5.92 (dd, *J*_1_ = 5.64 Hz, *J*_2_ = 12.44 Hz, 1H); 6.99–7.01 (d, *J* = 8.40 Hz, 2H); 7.17–7.19 (d, *J* = 8.80 Hz, 2H); 7.29–7.33 (t, 1H); 7.38–7.40 (d, *J* = 8.48 Hz, 1H); 7.48–7.54 (m, 4H); 7.61–7.63 (d, *J* = 8.28 Hz, 2H); 7.72–7.74 (d, *J* = 8.68 Hz, 2H); 7.88–7.94 (m, 4H); 8.29 (s, 1H); 11.43 (s, 1H). ^13^C-NMR (100 MHz, DMSO-*d*_6_): 163.09, 161.07, 153.53, 149.96, 146.85, 146.58, 133.93, 139.14, 133.41, 132.89, 129.66, 129.63, 129.37, 128.92, 128.22, 128.07, 127.86, 127.56, 126.98, 126.59, 124.92, 124.26, 123.18, 114.75, 112.44, 104.02, 63.17, 56.46, 55.73, 43.51. MS *m/z*: [M + H]^+^ 561.6 (C_34_H_26_F_2_N_4_O_2_). Anal. Calcd. for C_34_H_26_F_2_N_4_O_2_: C, 72.85; H, 4.67; N, 9.99;. Found: C, 70.13; H, 4.92; N, 9.59.

*(E)-4-(3-(3-Chlorophenyl)-5-(naphthalen-2-yl)-4,5-dihydro-1H-pyrazol-1-yl)-N′-(4-methoxybenzylidene)-benzohydrazide* (**H29**). Orange powder. Yield: 87.21%. m.p. 241–243 °C. ^1^H-NMR (400 MHz, DMSO-*d*_6_): 3.29–3.34 (dd, *J*_1_ = 5.48 Hz, *J*_2_ = 17.64 Hz, 1H); 3.79 (s, 3H); 4.01–4.08 (dd, *J*_1_ = 12.16 Hz, *J*_2_ = 17.48 Hz, 1H); 5.84–5.88 (dd, *J*_1_ = 5.52 Hz, *J*_2_ = 12.32 Hz, 1H); 6.98–7.00 (d, *J* = 8.48 Hz, 2H); 7.14–7.16 (d, *J* = 8.88 Hz, 2H); 7.38–7.44 (m, 2H); 7.49–7.51 (m, 2H); 7.60–7.62 (d, *J* = 7.12 Hz, 3H); 7.72–7.74 (d, *J* = 8.76 Hz, 2H); 7.80–7.82 (d, J = 7.80 Hz, 1H); 7.87–7.93 (m, 4H); 8.01 (s, 1H); 8.29 (s, 1H); 11.41 (s, 1H). ^13^C-NMR (100 MHz, DMSO-*d*_6_): 170.21, 163.07, 161.09, 149.72, 14690, 146.43, 139.52, 134.32, 133.32, 132.90, 131.37, 129.67, 129.40, 129.35, 128.28, 128.08, 127.56, 126.98, 126.66, 125.11, 124.24, 123.51, 114.75, 112.60, 63.20, 55.72, 43.42. MS *m/z*: [M + H]^+^ 560.0 (75%), [M + 2 + H]^+^ 562.0 (25%), (C_34_H_27_ClN_4_O_2_). Anal. Calcd. for C_34_H_27_ClN_4_O_2_: C, 73.05; H, 4.87; N, 10.02. Found: C, 72.91; H, 4.86; N, 9.99.

*(E)-4-(3-(4-Chlorophenyl)-5-(naphthalen-2-yl)-4,5-dihydro-1H-pyrazol-1-yl)-N′-(4-methoxybenzylidene)-benzohydrazide* (**H30**). Orange powder. Yield: 66.67%. m.p. 237–239 °C. ^1^H-NMR (400 MHz, DMSO-*d*_6_): 3.27–3.32 (dd, *J*_1_ = 5.44 Hz, *J*_2_ = 17.72 Hz, 1H); 3.79 (s, 3H); 4.01–4.06 (dd, *J*_1_ = 5.92 Hz, *J*_2_ = 17.48 Hz, 1H); 5.81–5.86 (dd, *J*_1_ = 5.72 Hz, *J*_2_ = 12.32 Hz, 1H); 6.98–7.00 (d, *J* = 8.48 Hz, 2H); 7.13–7.15 (d, *J* = 8.80 Hz, 2H); 7.38–7.40 (m, 1H); 7.49–7.54 (m, *J*_1_ = 4.00 Hz, *J*_2_ = 8.64 Hz, 4H); 7.61–7.63 (d, *J* = 8.28 Hz, 2H); 7.72–7.74 (d, *J* = 8.68 Hz, 2H); 7.83–7.93 (m, 6H); 8.30 (s, 1H); 11.42 (s, 1H). ^13^C-NMR (100 MHz, DMSO-*d*_6_): 170.81, 163.01, 161.08, 148.79, 146.89, 146.47, 139.73, 134.11, 133.39, 132.90, 131.27, 129.67, 129.40, 129.25, 128.20, 128.08, 127.56, 126.98, 126.63, 125.11, 124.24, 123.51, 114.75, 112.60, 63.40, 60.22, 55.72, 43.19. MS *m/z*: [M + H]^+^ 560.0 (75%), [M + 2 + H]^+^ 562.0 (25%), (C_34_H_27_ClN_4_O_2_). Anal. Calcd. for C_34_H_27_ClN_4_O_2_: C, 73.05; H, 4.87; N, 10.02. Found: C, 72.00; H, 4.95; N, 9.71.

*(E)-4-(3-(3,4-Dichlorophenyl)-5-(naphthalen-2-yl)-4,5-dihydro-1H-pyrazol-1-yl)-N'-(4-methoxy-benzylidene)benzohydrazide* (**H31**). Orange powder. Yield: 73.45%. m.p. 243–245 °C. ^1^H NMR (400 MHz, DMSO-*d*_6_): 3.32–3.37 (dd, *J*_1_ = 5.44 Hz, *J*_2_ = 17.92 Hz, 1H); 3.79 (s, 3H); 4.01–4.08 (dd, *J*_1_ = 12.60 Hz, *J*_2_ = 17.72 Hz, 1H); 5.85–5.90 (dd, *J*_1_ = 5.80 Hz, *J*_2_ = 12.36 Hz, 1H); 6.98–7.00 (d, *J* = 8.48 Hz, 2H); 7.15–7.17 (d, *J* = 8.80 Hz, 2H); 7.38–7.40 (d, *J* = 8.48 Hz, 1H); 7.49–7.51 (m, 2H); 7.61–7.63 (d, *J* = 8.20 Hz, 2H); 7.71–7.74 (m, 3H); 7.79–7.82 (m, 1H); 7.87–7.93 (m, 4H); 8.02 (s, 1H); 8.29 (s, 1H); 11.42 (s, 1H). ^13^C NMR (100 MHz, DMSO-*d*_6_): 163.08, 161.07, 149.89, 146.78, 146.47, 139.52, 134.32, 133.39, 132.87, 131.27, 129.67, 129.40, 129.25, 128.20, 128.08, 127.56, 126.98, 126.33, 125.11, 124.24, 123.51, 114.75, 112.60, 60.52, 55.72, 43.42. MS *m/z*: [M + H]^+^ 594.5 (75%), [M + 2 + H]^+^ 598.5 (25%), (C_34_H_26_Cl_2_N_4_O_2_). Anal. Calcd. for C_34_H_26_Cl_2_N_4_O_2_: C, 68.81; H, 4.42; N, 9.44. Found: C, 68.68; H, 4.40; N, 9.42.

*(E)-4-(3-(4-Bromophenyl)-5-(naphthalen-2-yl)-4,5-dihydro-1H-pyrazol-1-yl)-N′-(4-methoxybenzylidene)-benzohydrazide* (**H32**). Yellow powder. Yield: 70.90%. m.p. 238–240 °C. ^1^H-NMR (400 MHz, DMSO-*d*_6_): 3.26–3.32 (dd, *J*_1_ = 5.44 Hz, *J*_2_ = 17.80 Hz, 1H); 3.79 (s, 3H); 4.01–4.08 (dd, *J*_1_ = 11.96 Hz, *J*_2_ = 17.84 Hz, 1H); 5.81–5.85 (dd, *J*_1_ = 5.60 Hz, *J*_2_ = 12.20 Hz, 1H); 6.98–7.00 (d, *J* = 8.00 Hz, 2H); 7.13–7.15 (d, *J* = 8.48 Hz, 2H); 7.38–7.40 (d, *J* = 8.32 Hz, 1H); 7.49–7.51 (m, 2H); 7.61–7.67 (m, 4H); 7.72–7.77 (m, 4H); 7.87–7.93 (m, 4H); 8.29 (s,1H); 11.41 (s, 1H). ^13^C-NMR (100 MHz, DMSO-*d*_6_): 163.00, 161.08, 148.86, 146.89, 146.47, 139.71, 133.38, 132.90, 131.60, 129.67, 129.40, 128.94, 128.43, 128.25, 127.55, 126.98, 126.63, 125.11, 124.23, 123.53, 122.83, 114.75, 112.61, 63.40, 55.73, 43.12. MS *m/z*: [M + H]^+^ 604.5 (50%), [M + 2 + H]^+^ 606.5 (50%), (C_34_H_27_BrN_4_O_2_). Anal. Calcd. for C_34_H_27_BrN_4_O_2_: C, 67.67; H, 4.51; N, 9.28. Found: C, 66.49; H, 6.35; N, 7.86.

### 3.3. Biological Assays

#### 3.3.1. Antiproliferation Activity

IC_50_ values of the test compounds against A549, MCF-7, Hela and HepG2 cells were obtained from State Key Laboratory of Pharmaceutical Biotechnology, Nanjing University, and were determined by a standard (MTT)-based colorimetric assay (Beyotime Inst. Biotech., Nanjing, China). Tumor cell lines were grown to mid-log phase and then diluted to 5 × 10^3^–1 × 10^4^ cells/mL making sure they were seeded in 96-well plates (Philas, Nanjing, China) at a density of 5 × 10^3^–1 × 10^4^ cells per well. The outer wells containing 1 × PBS solution of the plate are not used for the assay to avoid the possibility of edge effects. The plate was incubated at 37 °C in a 5% CO_2_ incubator in DMEM containing 10% fetal bovine serum (FBS, Gibco, Waltham, MA, USA) for 24 h. Then, we added a 100-μL series concentration of drug-containing medium into the wells to maintain the final concentration of drug as 100, 10, 1 and 0.1 μmol/L. After 12 h, cell survival was determined by the addition of a MTT (3-(4,5-dimethylthiazol-2-yl)-2,5-diphenyl trtrazolium bromide) solution (10 μL of 5 mg/mL MTT in 1 × PBS). We waited for 4 h proliferation, discarded the medium and quickly added 150 μL DMSO per well, shaking the plates well for 10 min to ensure complete dissolution. Optical absorbance was measured at 570 nm on a microplate reader (Bio-Rad, Hercules, CA, USA). Survival ratios are expressed in percentages with respect to untreated cells. The experiments were replicated at least three times to verify the methodology reproducibility when using the above-mentioned conditions. The results were summarized in Table 2.

#### 3.3.2. Kinase Assay

EGFR tyrosine kinase activity was determined by an enzyme linked-immunosorbent assay (ELISA) in 96-well plates precoated in a 10 mL reaction volume including 4 mL diluted test compounds, 4 mL substrate and 2 mL ATP. After incubation for 1 h at 37 °C, the reactions were stopped with 10 mL 2% (*v*/*v*) H_3_PO_4_. The plates were then aspirated and washed twice with 200 mL 0.9% (*w*/*v*) NaCl, and incorporation of Pi was determined with an LX300 Epson Diagnostic microplate reader. The residual kinase activities for each concentration of compound and the compound IC_50_ values were expressed in percentages with respect to untreated cells. The experiments were replicated at least three times to verify the methodology reproducibility when using the above-mentioned conditions. The results were summarized in Table 3.

#### 3.3.3. Cytotoxicity Assay

The cytotoxic activity in vitro was measured by the colorimetric MTT assay. Cells were incubated in a 96-well plate at a density of 10^5^ cells per well with various concentrations of compounds (160, 40, 10, 2.5, 0.25 μM) in distilled 10% DMSO (10 μL) for 24 h. For the cytotoxicity assay, 20 μL of MTT (5 mg/mL) was added per well 4 h before the end of the incubation. After removing the supernatant, 200 μL DMSO was added to dissolve the formazan crystals. The absorbance at λ 570 nm was read on an LX300 Epson Diagnostic microplate reader, untreated cells were used as negative controls. The results are summarized in Table 4.

#### 3.3.4. Cell Apoptosis Assay by Flow Cytometry

For Annexin V/PI assays, HeLa cells were stained with Annexin VFITC and PI and then monitored for apoptosis by flow cytometry. Briefly, 5 × 10^3^ cells were seeded in 6-well plates for 24 h and then were treated with **H20** (2.0, 4.0, 6.0, 8.0 μM) for 24 h. Then, cells were collected and washed twice with PBS and stained with 5 μL Annexin V-FITC and 5 μL PI (5 μg/mL) in 1× binding buffer (10 mM HEPES, pH 7.4, 140 mM NaOH, 2.5 mM CaCl_2_) for 15 min at room temperature in the dark. Apoptotic cells were quantified using a BD Accuri C_6_ Flow Cytometer (Becton, Dickinson and Company, Clifton, NJ, USA). Statistical analysis was done using Flowjo 7.6.1 software. Both early apoptotic (AnnexinV-positive, PI-negative) and late apoptotic (double positive of Annexin V and PI) cells were detected.

### 3.4. Docking Simulations

The crystal structures of the proteins complex were retrieved from the RCSB Protein Data Bank (PDB code: 1M17). Then, three-dimensional structures of the aforementioned compounds were constructed using Chem 3D Ultra 14.0 software (Cambridge Soft Corporation, Boston MA, USA), then they were energetically minimized by using MMFF94 with 5000 iterations and minimum RMS gradient of 0.10. Molecular docking of compound **H20** into the three-dimensional EGFR complex structure was carried out using the Discovery Studio (Discovery Studio 4.5, Accelrys, Co. Ltd., Beijing, China) software as implemented through the graphical user interface DS-CDOCKER protocol. All bound waters and ligands were eliminated from the protein and the polar hydrogen was added to the proteins. Briefly, we define the EGFR complex as a receptor, then perform simulation of the compound into the ATP binding site in EGFR by replacing ATP molecule with our compound **H20**.

## 4. Conclusions

A series of benzohydrazide derivatives **H1**–**H32** containing dihydropyrazoles were synthesized and evaluated in vitro for anticancer activities against the A549, MCF-7, HeLa and HepG2 cell lines, EGFR inhibitory activities, as well as MTT assays. These compounds exhibited potent EGFR inhibitory activities and antiproliferative activities against the above four cell lines. Among them, compound **H20** showed the most potent EGFR inhibition activities (IC_50_ = 0.08 μM) and anticancer activities against A549, MCF-7, HeLa and HepG2 (IC_50_ = 0.46, 0.29, 0.15 and 0.21 μM, respectively). Docking simulation of the binding model of compound **H20** with EGFR indicated that conventional hydrogen bonds and Van der Waals interactions with the protein residues in the ATP binding site might play a crucial role in its EGFR inhibition and antiproliferative activities. Therefore, compound **H20** may be developed as a potential antitumor agent.

## Figures and Tables

**Table 1:** Structures of compounds **H1**–**H32**.

Compound	R_1_	R_2_	R_3_	R_4_	R_5_	R_6_
**H1**	H	H	H	H	OCH_3_	H
**H2**	CH_3_	H	H	H	OCH_3_	H
**H3**	H	CH_3_	H	H	OCH_3_	H
**H4**	H	H	CH_3_	H	OCH_3_	H
**H5**	H	H	CH_2_CH_3_	H	OCH_3_	H
**H6**	H	H	OCH_3_	H	OCH_3_	H
**H7**	H	OCH_3_	OCH_3_	OCH_3_	OCH_3_	F
**H8**	H	H	OCH_2_CH_3_	H	OCH_3_	H
**H9**	F	H	H	H	OCH_3_	H
**H10**	H	F	H	H	OCH_3_	H
**H11**	H	H	F	H	OCH_3_	H
**H12**	H	F	H	F	OCH_3_	H
**H13**	H	Cl	H	H	OCH_3_	H
**H14**	H	H	Cl	H	OCH_3_	H
**H15**	H	Cl	Cl	H	OCH_3_	H
**H16**	H	H	Br	H	OCH_3_	H
**H17**	H	H	H	H	H	OCH_3_
**H18**	CH_3_	H	H	H	H	OCH_3_
**H19**	H	CH_3_	H	H	H	OCH_3_
**H20**	H	H	CH_3_	H	H	OCH_3_
**H21**	H	H	CH_2_CH_3_	H	H	OCH_3_
**H22**	H	H	OCH_3_	H	H	OCH_3_
**H23**	H	OCH_3_	OCH_3_	OCH_3_	F	OCH_3_
**H24**	H	H	OCH_2_CH_3_	H	H	OCH_3_
**H25**	F	H	H	H	H	OCH_3_
**H26**	H	F	H	H	H	OCH_3_
**H27**	H	H	F	H	H	OCH_3_
**H28**	H	F	H	F	H	OCH_3_
**H29**	H	Cl	H	H	H	OCH_3_
**H30**	H	H	Cl	H	H	OCH_3_
**H31**	H	Cl	Cl	H	H	OCH_3_
**H32**	H	H	Br	H	H	OCH_3_

**Table 2:** In vitro antiproliferative activities (IC_50_, μM ^a^) of all compounds against tumor cell lines.

Compounds	A549 ^b^	MCF-7 ^b^	HeLa ^b^	HepG2 ^b^
**H1**	14.52	20.96	22.47	28.76
**H2**	1.41	1.52	0.60	0.67
**H3**	3.02	2.89	2.45	3.54
**H4**	0.43	0.69	0.45	0.51
**H5**	5.49	7.72	8.71	6.16
**H6**	8.32	10.17	13.16	11.45
**H7**	78.32	95.32	>100	>100
**H8**	10.71	15.87	17.08	19.75
**H9**	50.65	70.36	63.78	76.48
**H10**	58.59	80.12	84.12	92.50
**H11**	45.16	60.79	56.21	60.86
**H12**	41.48	55.64	50.34	52.30
**H13**	35.25	51.28	46.38	45.37
**H14**	30.14	42.46	37.43	40.20
**H15**	26.57	35.24	33.62	38.93
**H16**	21.78	31.85	26.51	34.19
**H17**	13.30	18.39	20.73	26.67
**H18**	1.22	1.63	0.78	0.56
**H19**	2.24	2.45	1.19	3.26
**H20**	0.46	0.29	0.15	0.21
**H21**	4.54	6.33	7.93	5.06
**H22**	7.12	9.69	12.31	10.54
**H23**	75.66	93.23	94.10	89.47
**H24**	9.92	13.79	15.28	18.56
**H25**	48.55	65.53	61.87	71.10
**H26**	56.39	77.49	85.15	80.07
**H27**	44.11	58.98	54.22	62.94
**H28**	39.42	56.14	47.67	48.06
**H29**	33.25	50.88	42.95	40.99
**H30**	28.64	40.40	35.34	38.20
**H31**	24.77	34.44	31.82	36.33
**H32**	19.71	30.25	25.55	32.91
**Erlotinib**	0.20	0.12	0.17	0.10

^a^ Antiproliferation activity was measured using the MTT assay. Values are the average of six independent experiments run in triplicate. Variation was generally 5%–10%; ^b^ Cancer cells kindly supplied by State Key Laboratory of Pharmaceutical Biotechnology, Nanjing University.

**Table 3:** Inhibition activities (IC_50_, μM ^a^) of all compounds against epidermal growth factor receptor (EGFR).

Compounds	IC_50_, μM ^a^	Compounds	IC_50_, μM ^a^
**H1**	8.47	H17	7.89
**H2**	0.65	H18	0.48
**H3**	2.12	H19	1.25
**H4**	0.23	H20	0.08
**H5**	4.31	H21	3.56
**H6**	5.96	H22	5.07
**H7**	26.94	H23	24.58
**H8**	7.20	H24	6.65
**H9**	16.20	H25	15.33
**H10**	19.95	H26	18.55
**H11**	14.10	H27	13.78
**H12**	12.81	H28	12.34
**H13**	11.86	H29	11.24
**H14**	10.33	H30	10.02
**H15**	9.87	H31	9.66
**H16**	9.01	H32	8.64
**Erlotinib**	0.03		

^a^ EGFR inhibitory activity was measured by the solid-phase ELISA assay. Values are the average of six independent experiments run in triplicate. Variation was generally 5%–10%.

**Table 4:** The median cytotoxic concentration (CC_50_, μM ^a^) data of tested compounds

Compounds	CC_50_, μM ^a^	Compounds	CC_50_, μM ^a^
**H1**	98.56	H17	94.17
**H2**	87.45	H18	90.64
**H3**	92.32	H19	92.58
**H4**	98.83	H20	89.86
**H5**	92.50	H21	82.61
**H6**	81.75	H22	84.67
**H7**	92.93	H23	85.76
**H8**	79.69	H24	81.29
**H9**	83.43	H25	85.60
**H10**	95.79	H26	94.30
**H11**	86.52	H27	89.42
**H12**	80.24	H28	82.16
**H13**	93.35	H29	94.37
**H14**	90.14	H30	88.54
**H15**	88.23	H31	84.77
**H16**	81.87	H32	83.63
**Erlotinib**	89.32		

^a^ The cytotoxicity of each compound was expressed as the concentration of compound that reduced cell viability to 50% (CC_50_).

## Supplementary Materials

Supplementary materials can be accessed at: http://www.mdpi.com/1420-3049/21/8/1012/s1.
