# Supplementary material for: Design, Synthesis and Biological Evaluation of Benzohydrazide Derivatives Containing Dihydropyrazoles as Potential EGFR Kinase Inhibitors
Source: Molecules. 2016 Aug 3;21(8):1012. doi: 10.3390/molecules21081012 (PMC6273578; doi:10.3390/molecules21081012)
Supplement: Supplementary file 1 [file molecules-21-01012-s001.pdf]

Supplementary Materials: Design, Synthesis and Biological Evaluation of Benzohydrazide Derivatives Containing Dihydropyrazole as Potential EGFR Kinase Inhibitors

Hai-Chao Wang, Xiao-Qiang Yan, Tian-Long Yan, Hong-Xia Li, Zhong-Chang Wang and Hai-Liang Zhu

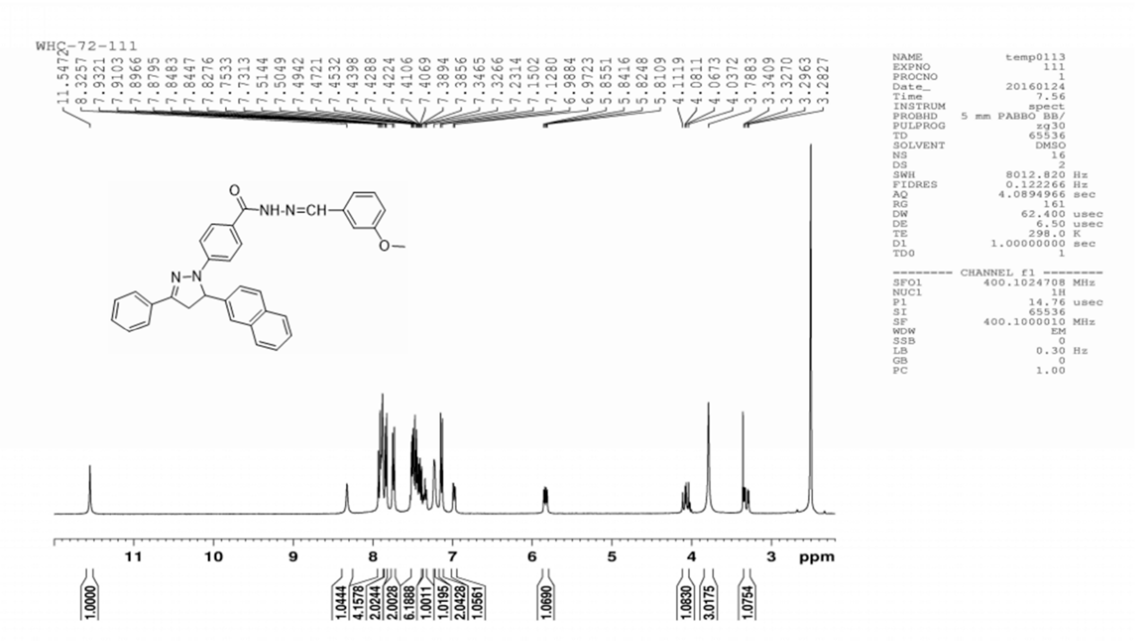

Figure S1. H1-^{1}H-NMR.

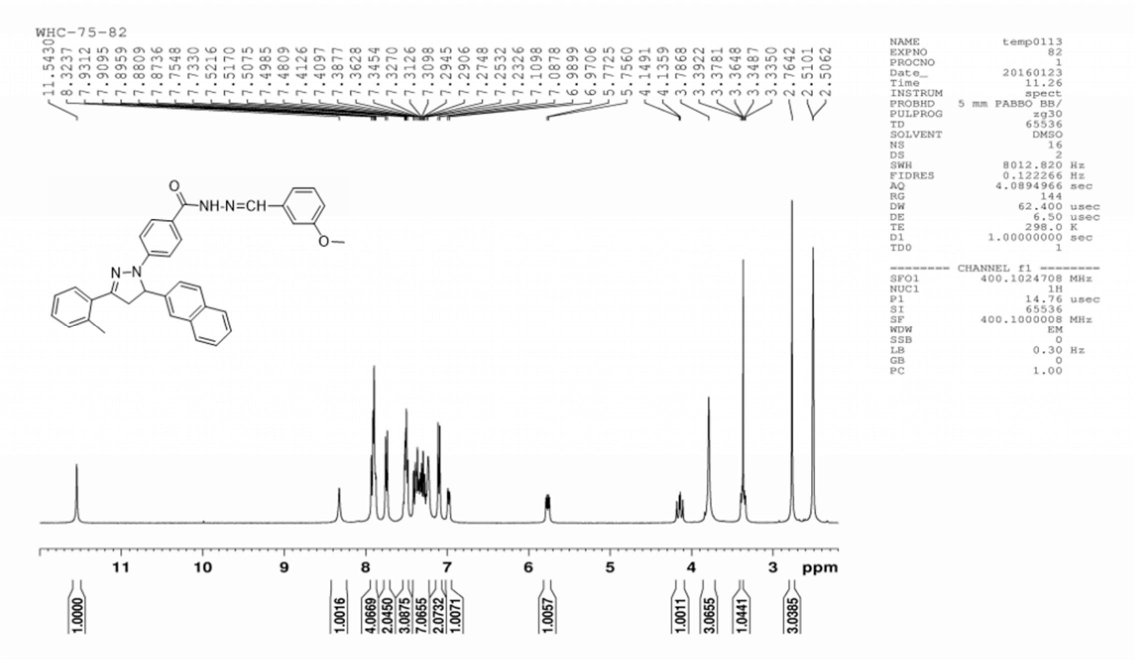

Figure S2. H2-^{1}H-NMR.

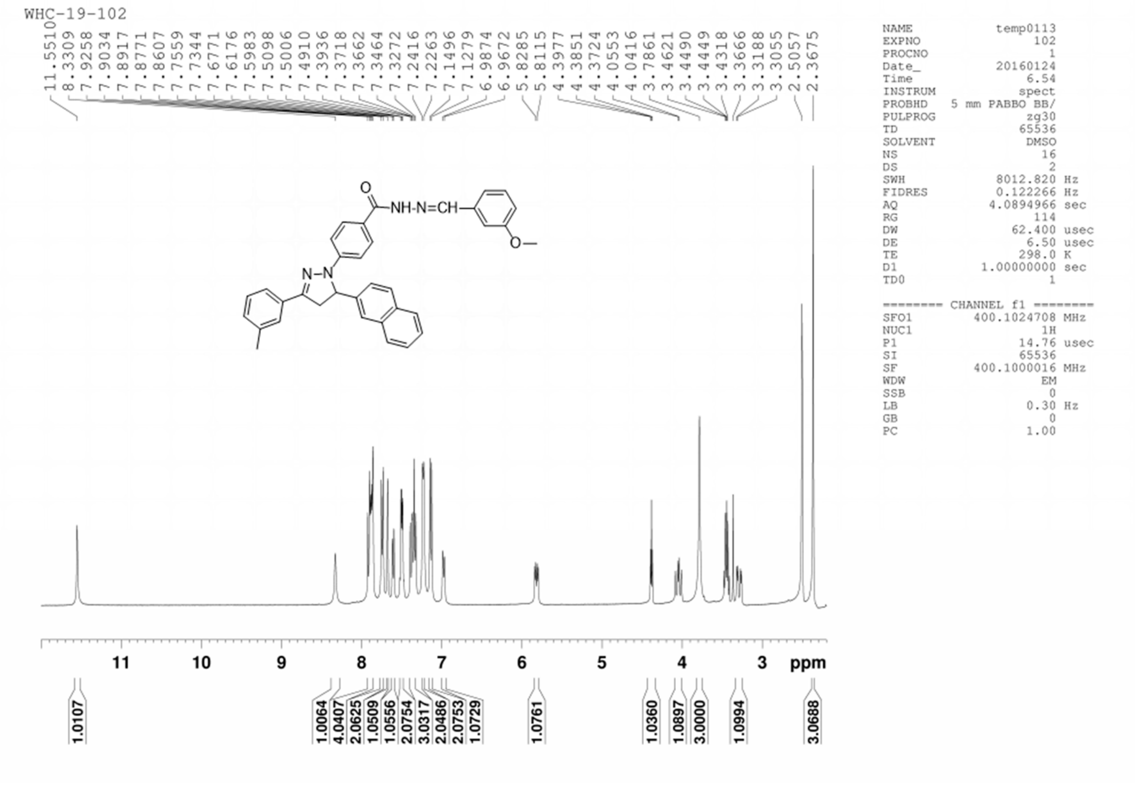

Figure S3. H3-¹H-NMR.

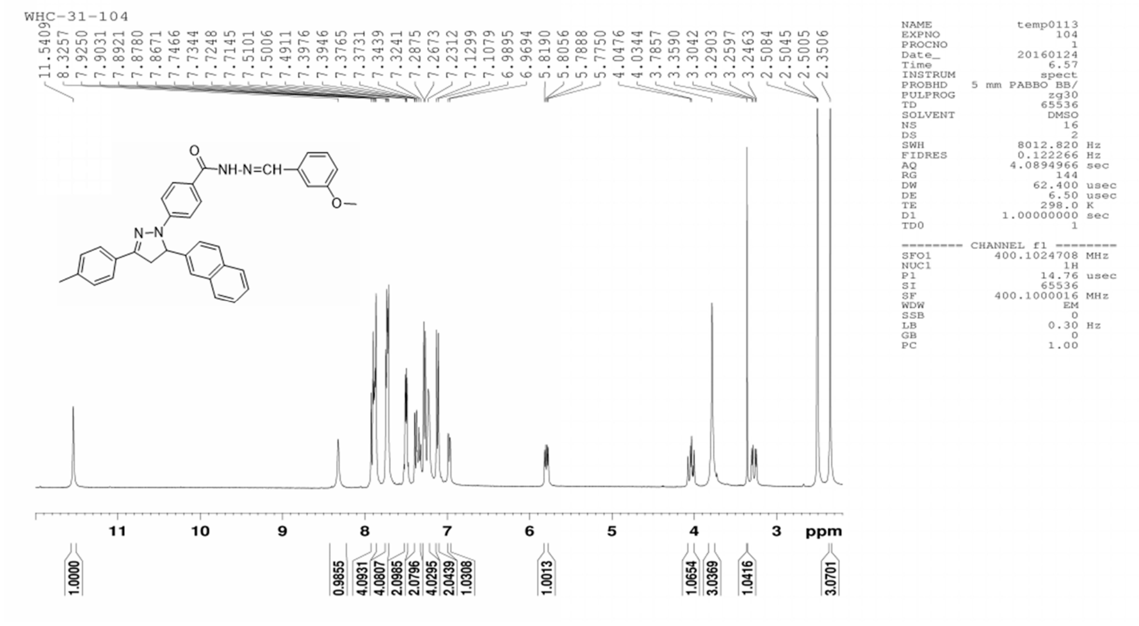

Figure S4. H4-¹H-NMR.

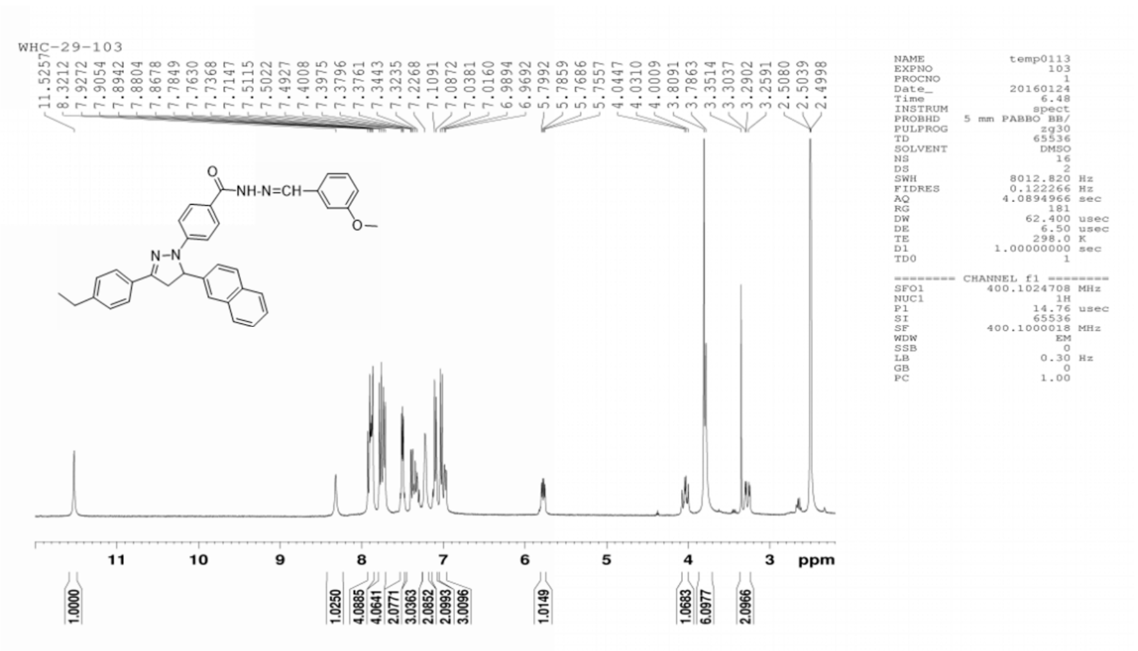

Figure S5. H5-^{1}H-NMR.

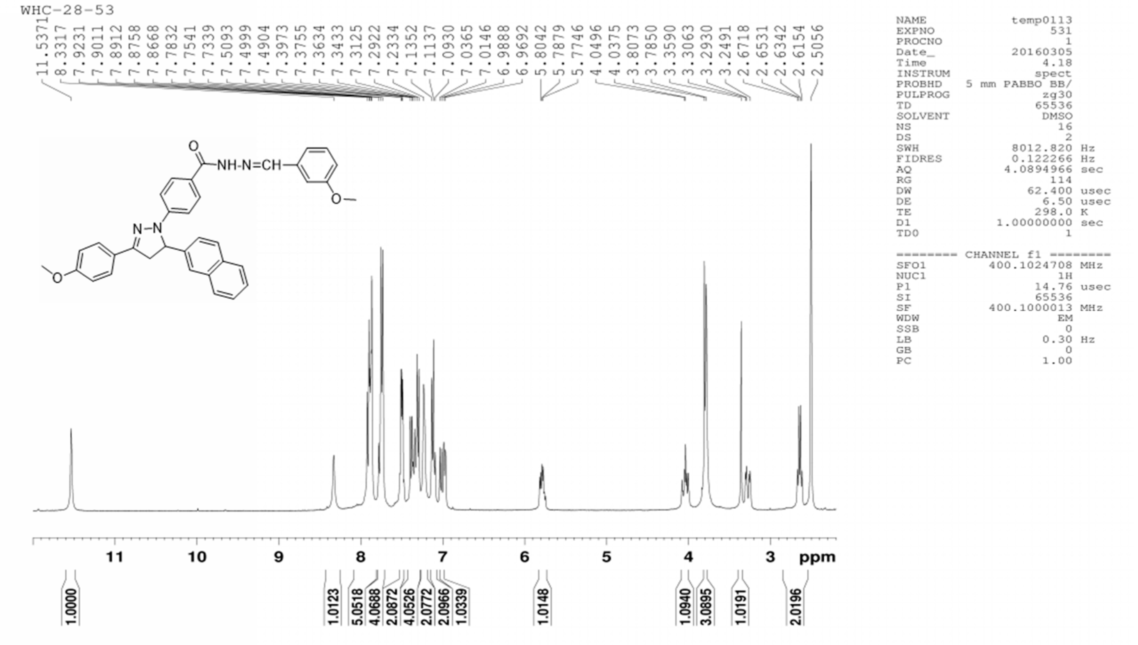

Figure S6. H6-^{1}H-NMR.

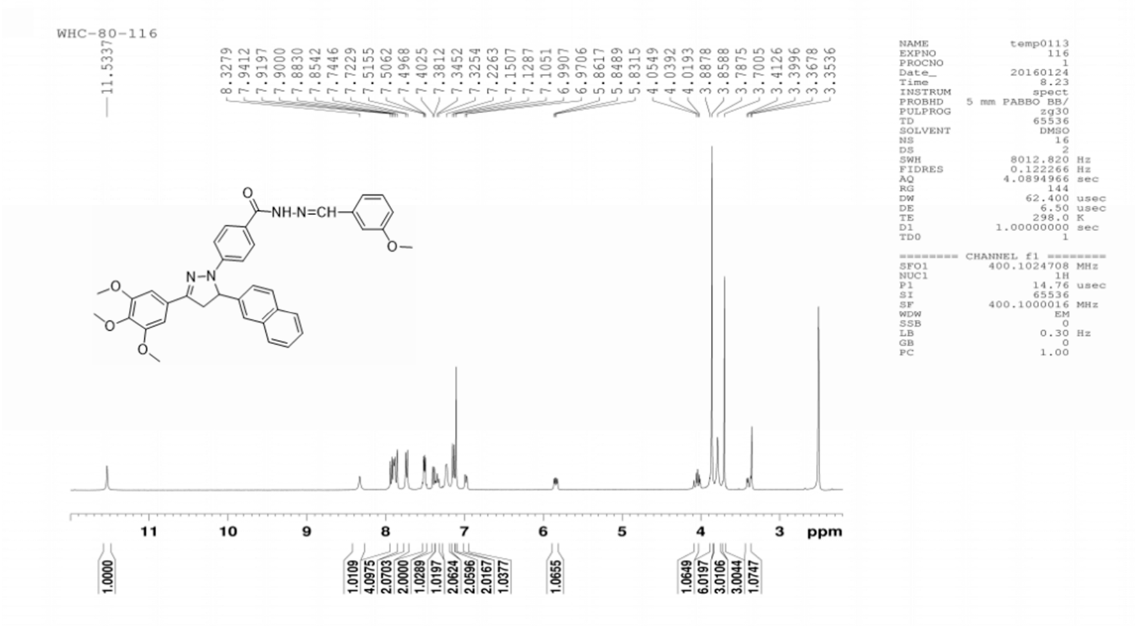

Figure S7. H7-¹H-NMR.

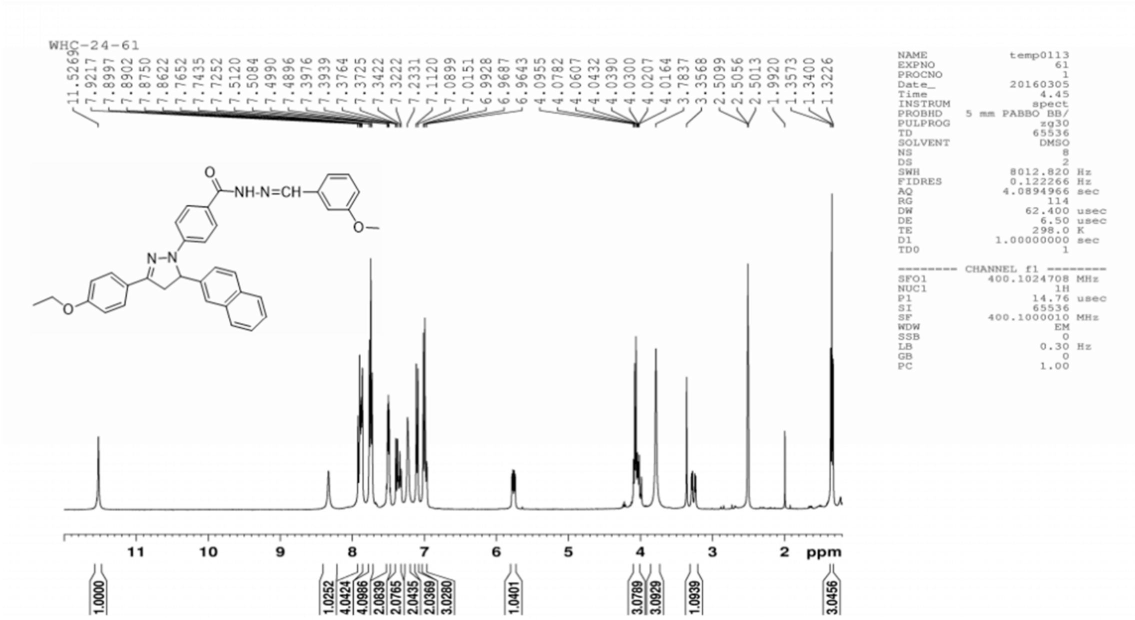

Figure S8. H8-¹H-NMR.

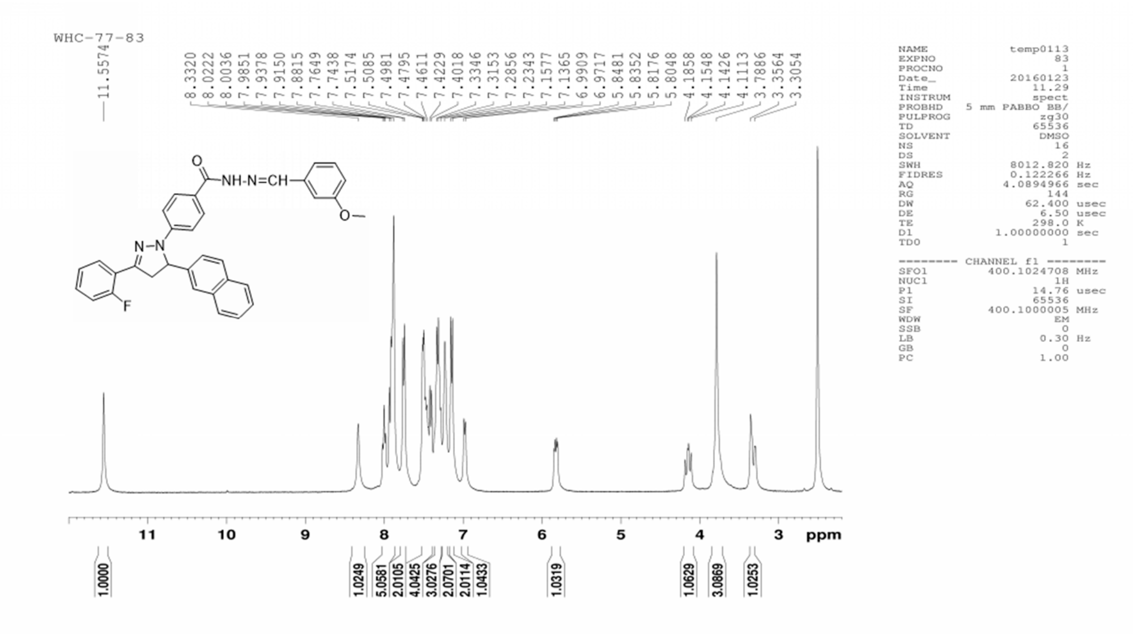

Figure S9. H9-^{1}H-NMR.

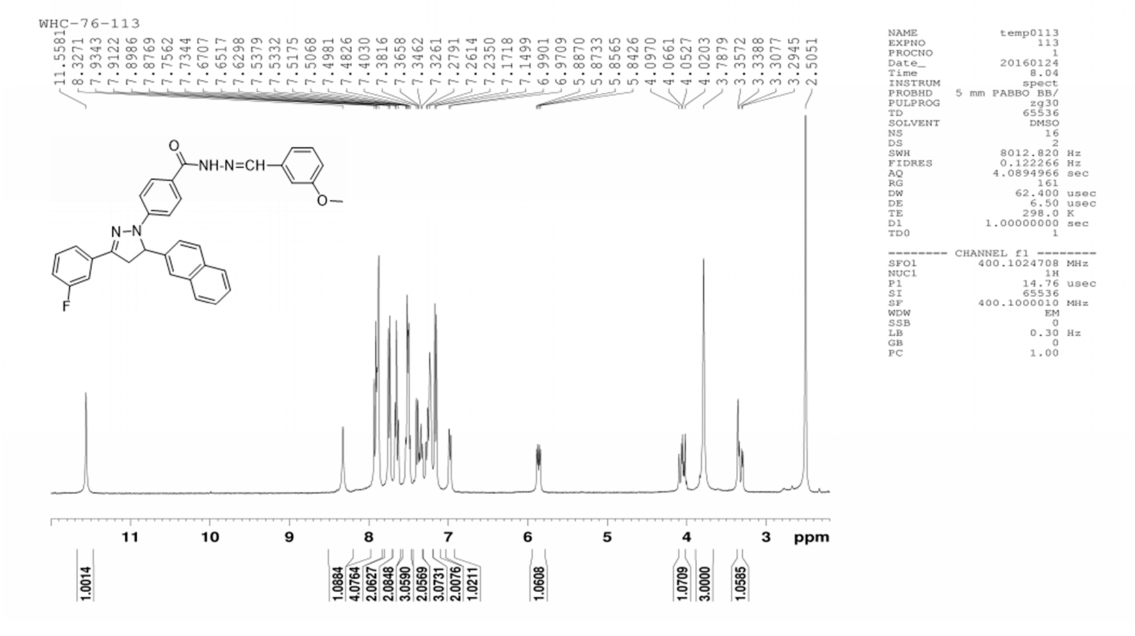

Figure S10. H10-^{1}H-NMR.

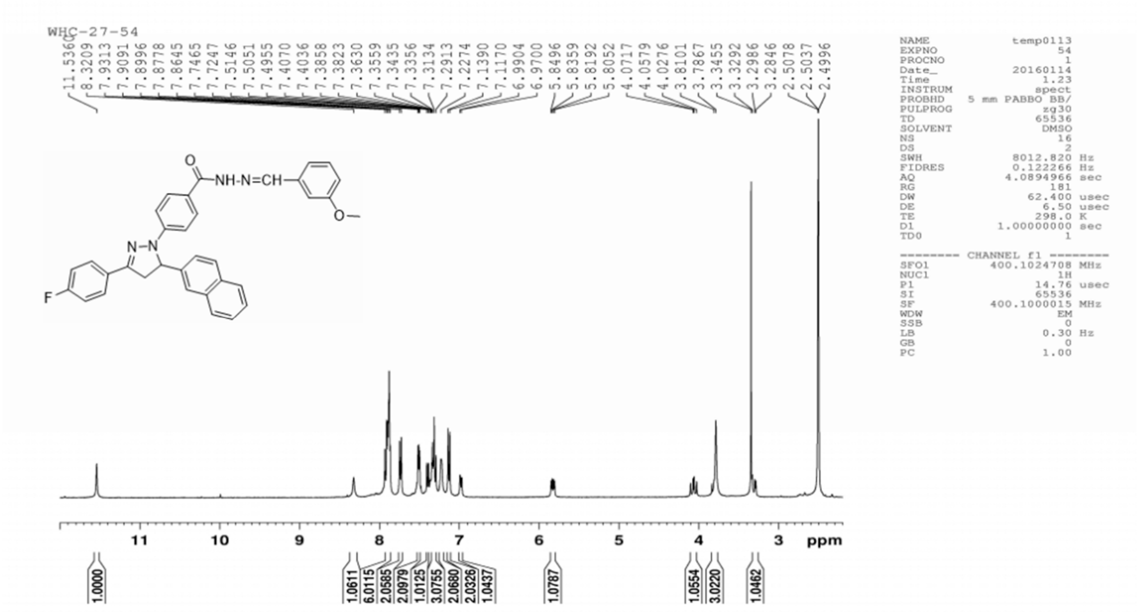

Figure S11. H11-^{1}H-NMR.

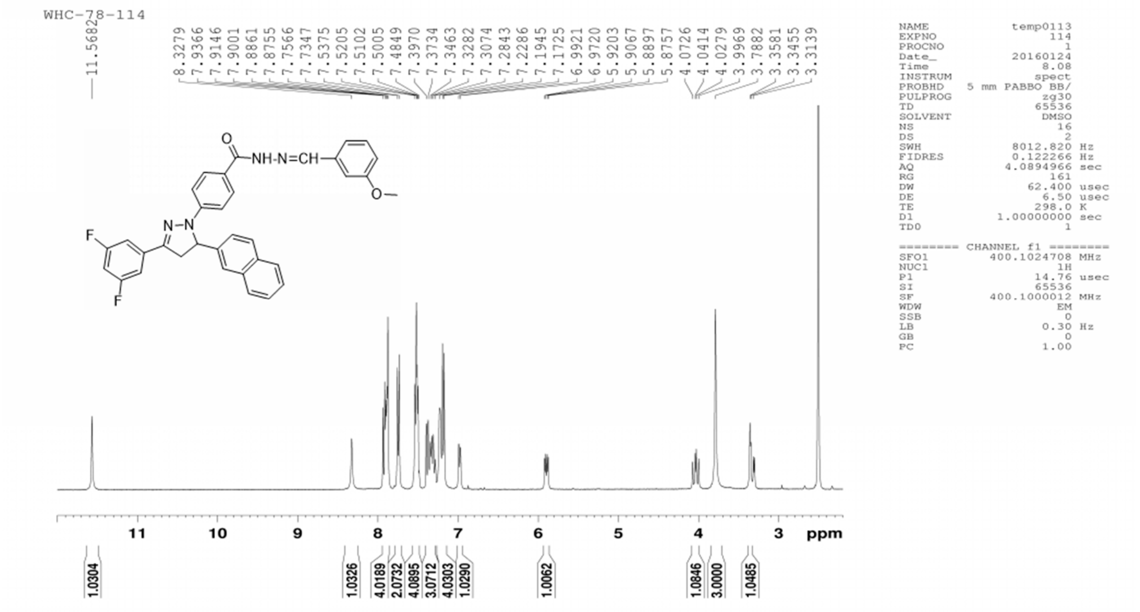

Figure S12. H12-^{1}H-NMR.

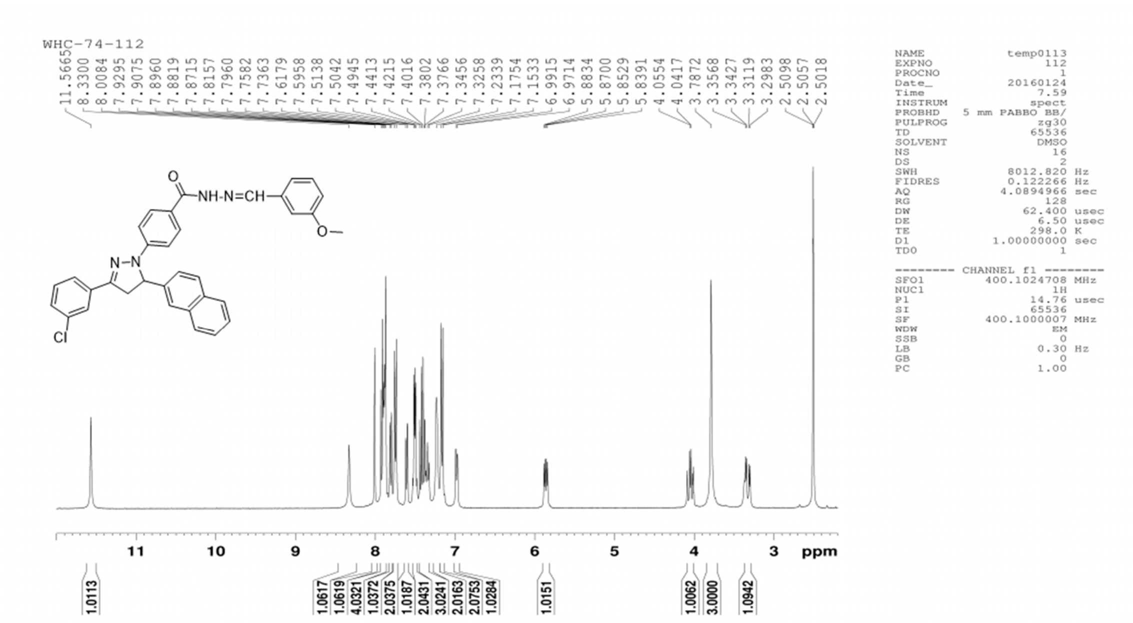

Figure S13. H13-¹H-NMR.

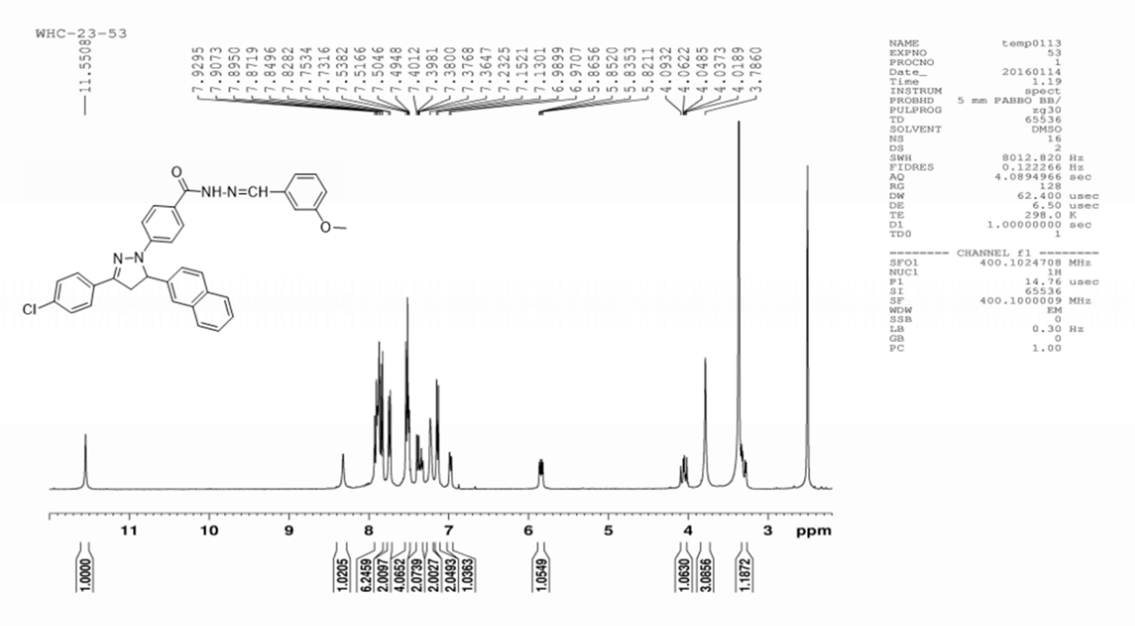

Figure S14. H14-¹H-NMR.

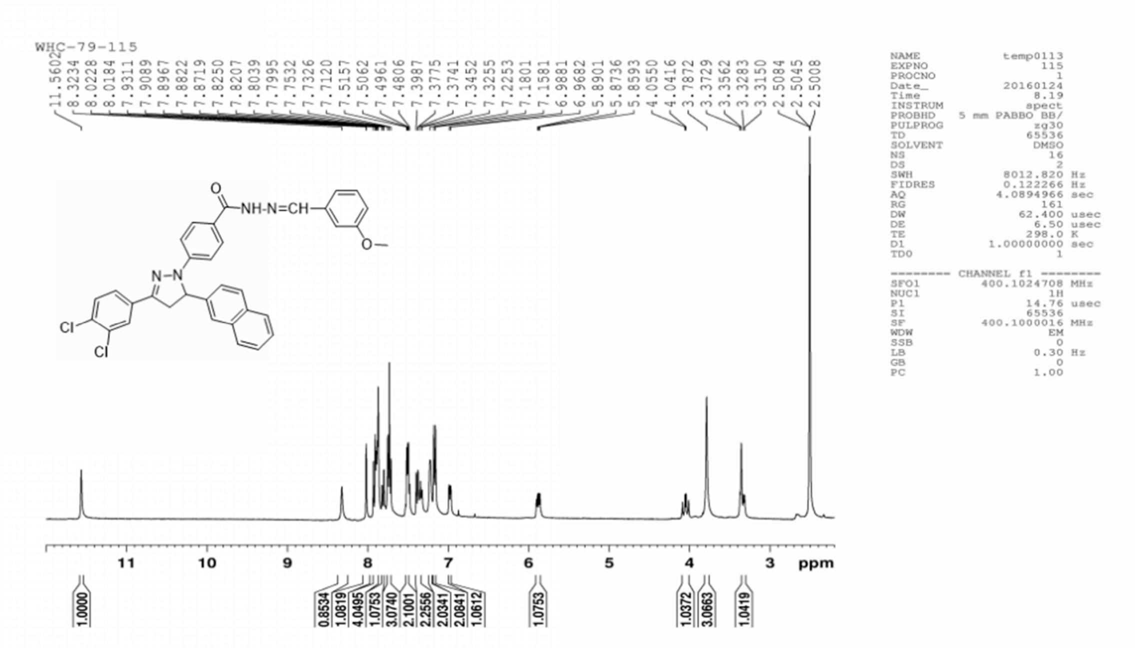

Figure S15. H15-^{1}H-NMR.

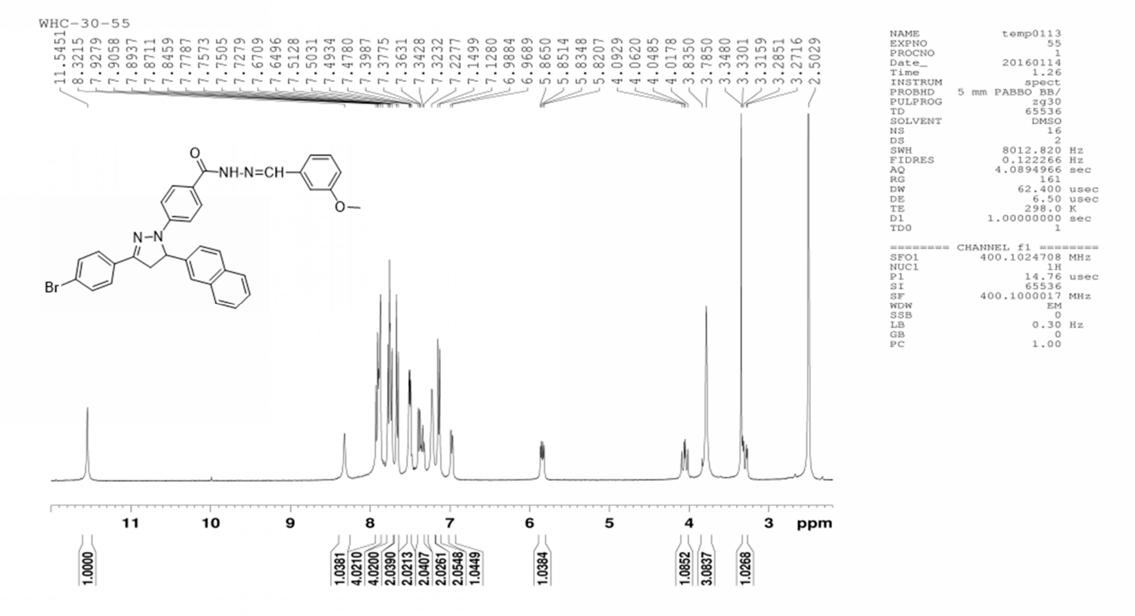

Figure S16. H16-^{1}H-NMR.

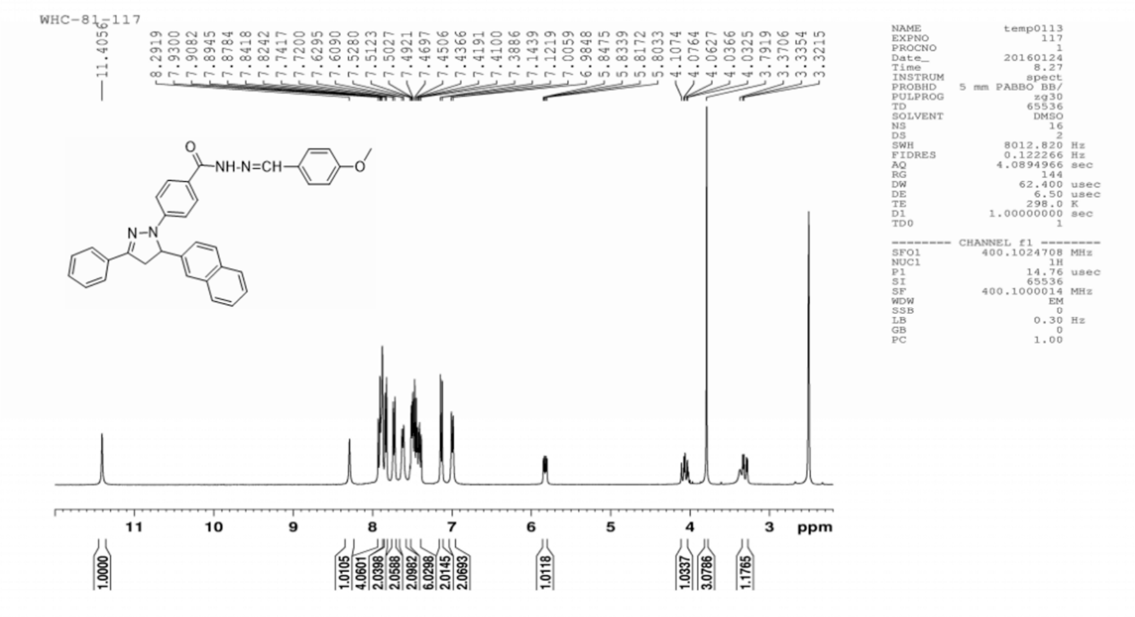

Figure S17. H17-¹H-NMR.

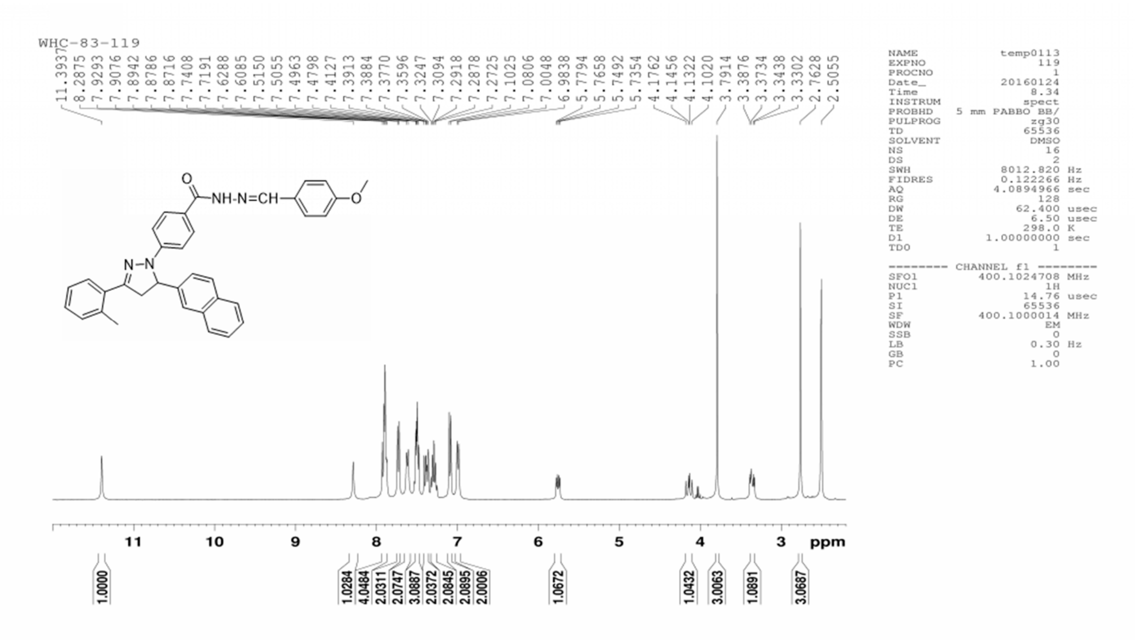

Figure S18. H18-¹H-NMR.

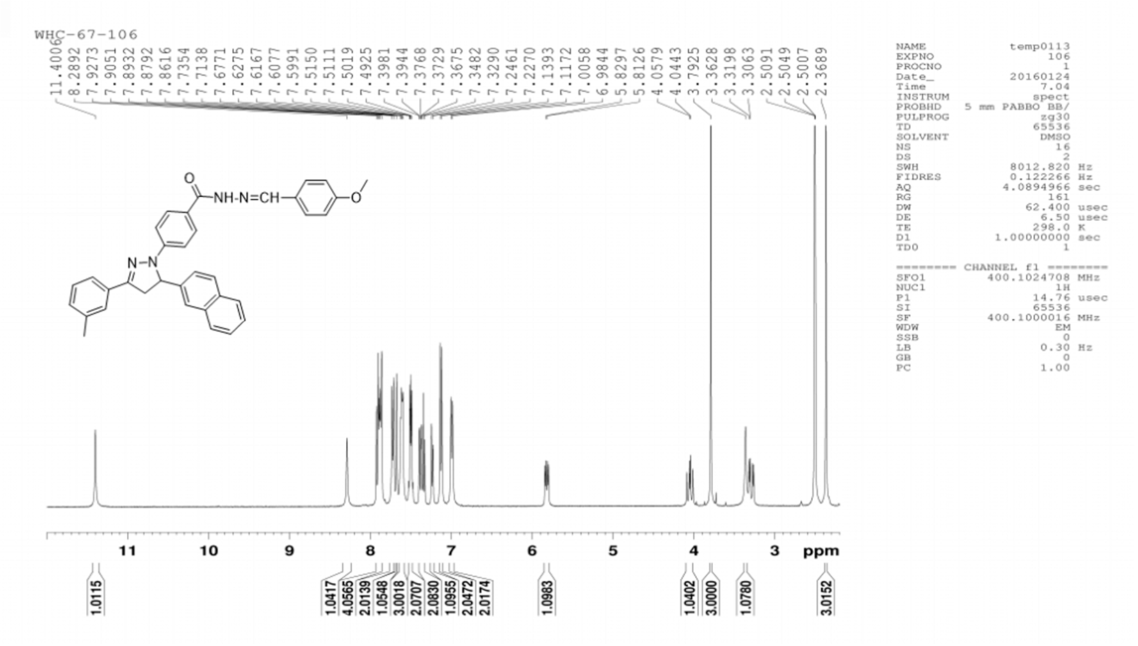

Figure S19. H19-^{1}H-NMR.

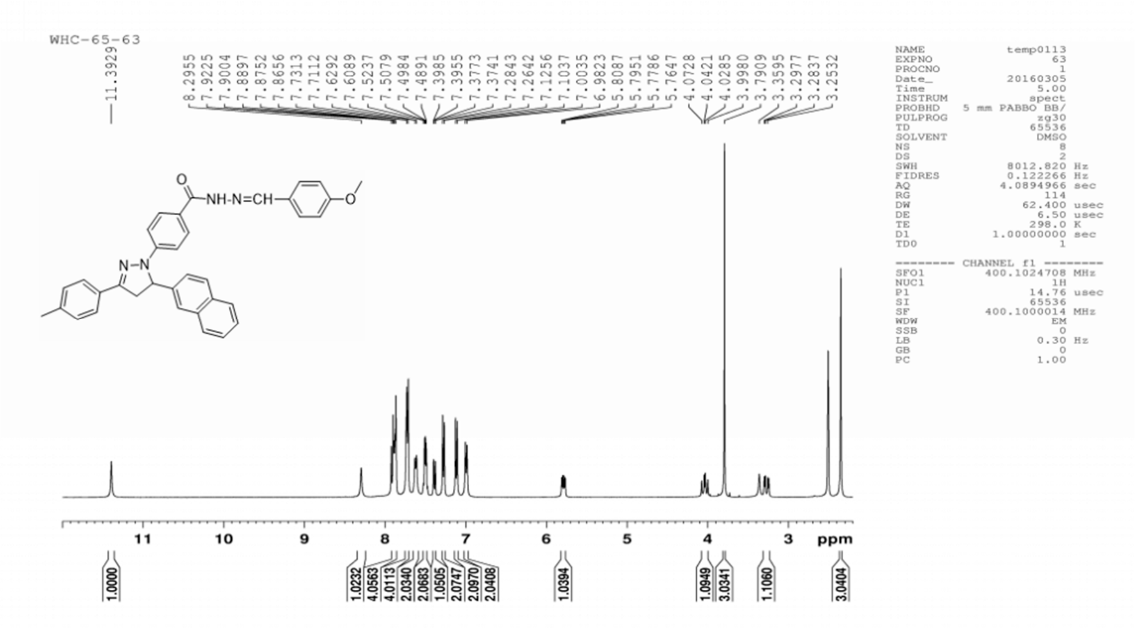

Figure S20. H20-^{1}H-NMR.

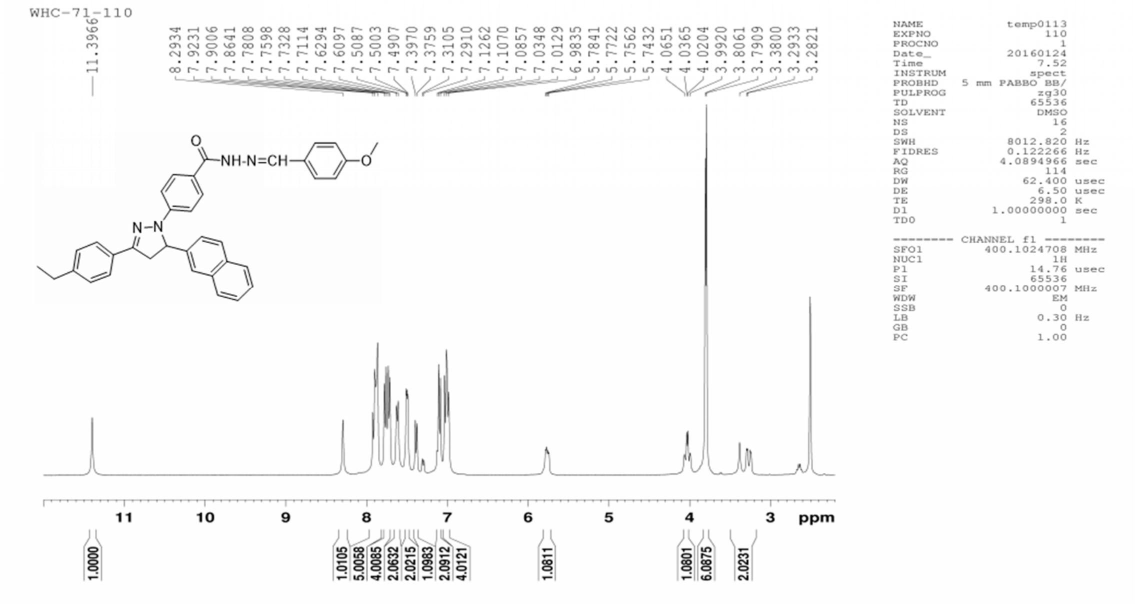

Figure S21. H21-^{1}H-NMR.

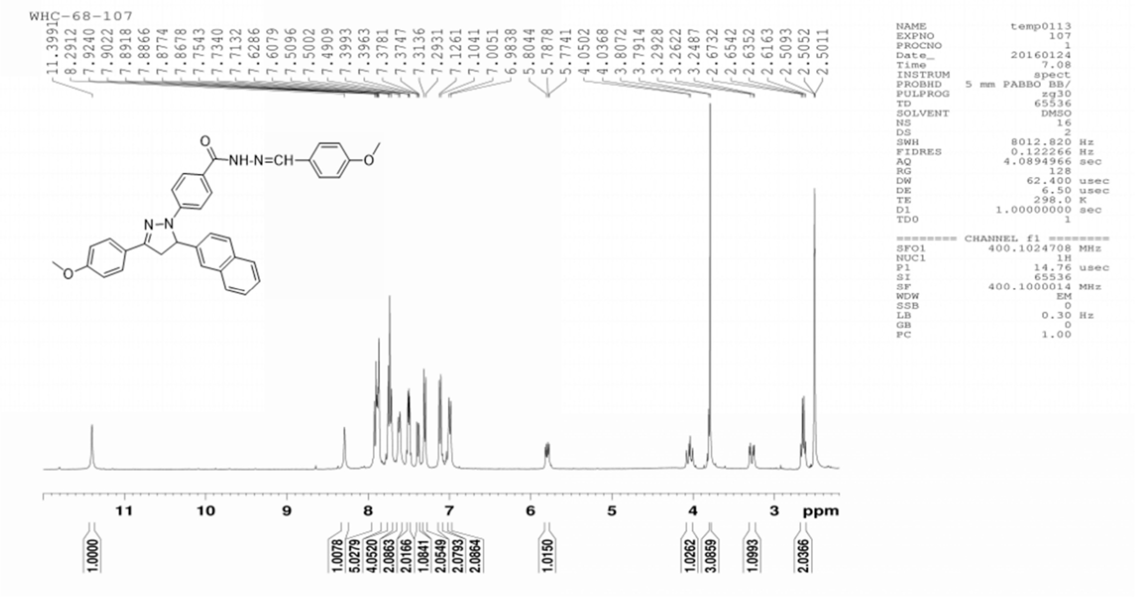

Figure S22. H22-^{1}H-NMR.

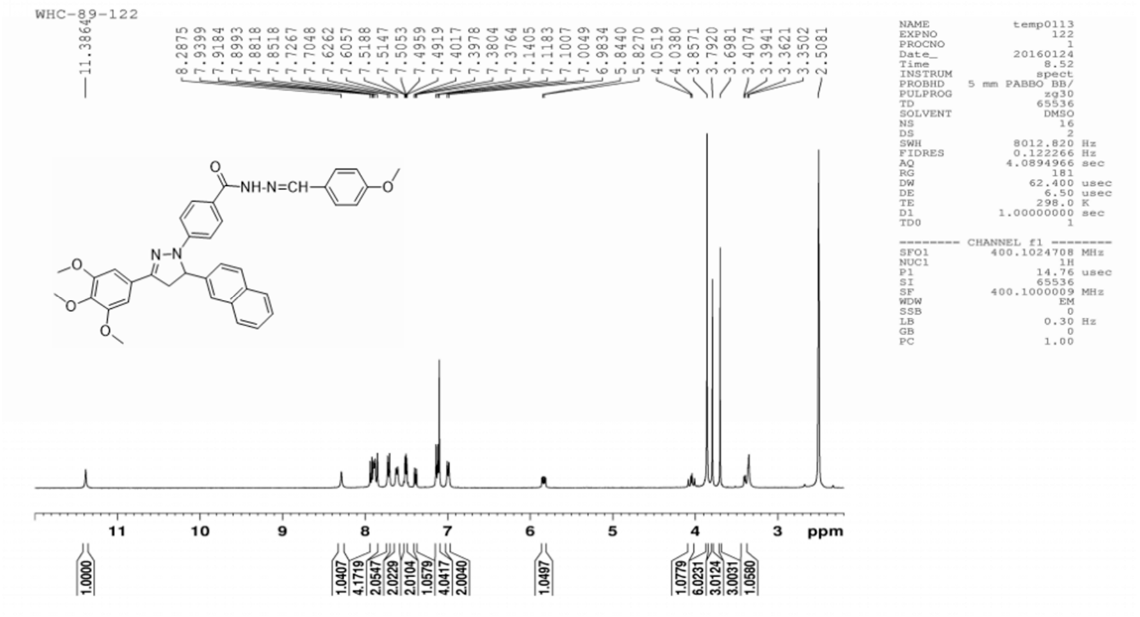

Figure S23. H23-^{1}H-NMR.

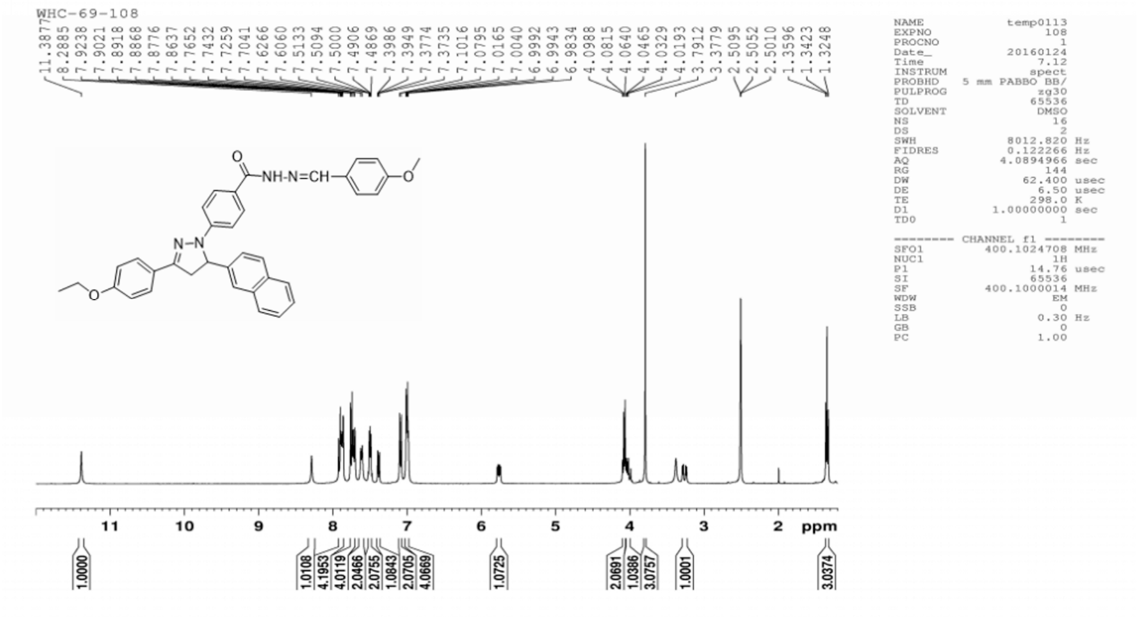

Figure S24. H24-^{1}H-NMR.

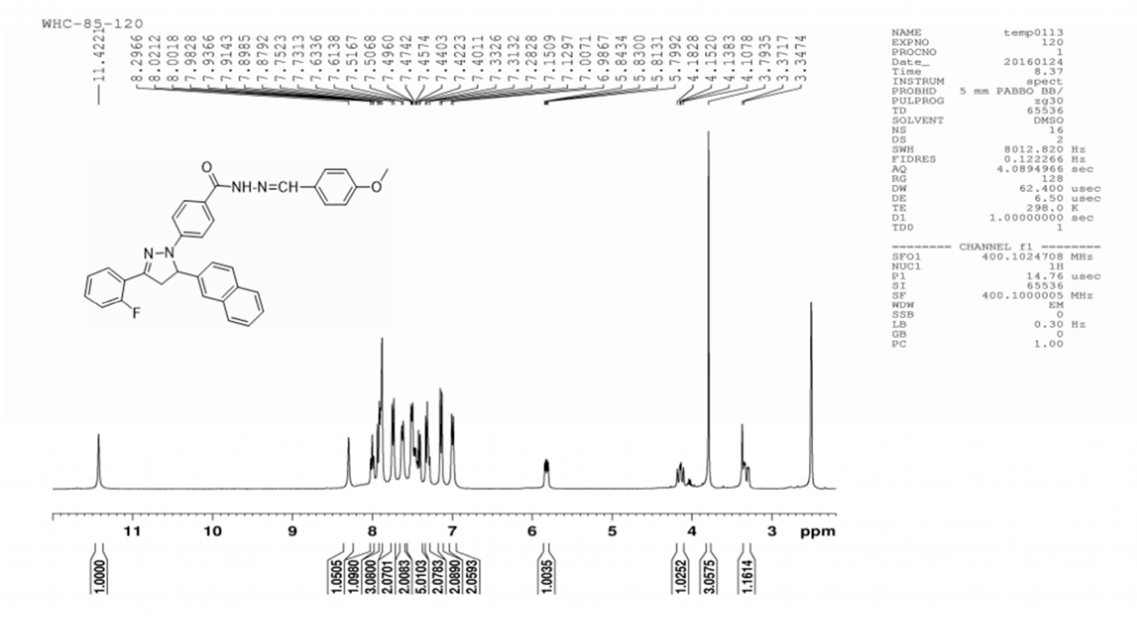

Figure S25. H25-^{1}H-NMR.

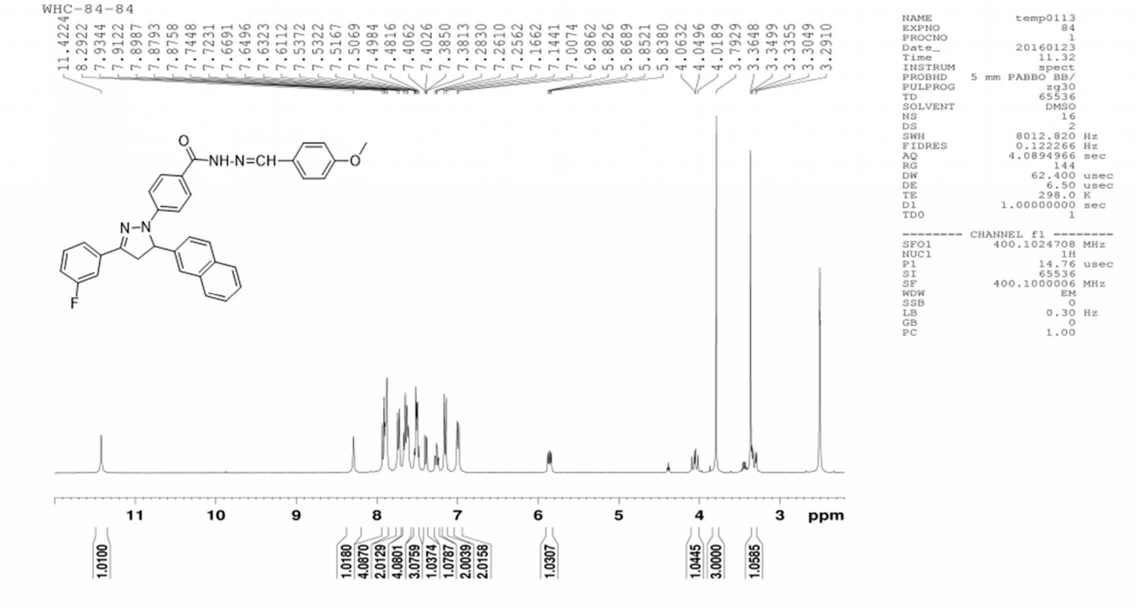

Figure S26. H26-^{1}H-NMR.

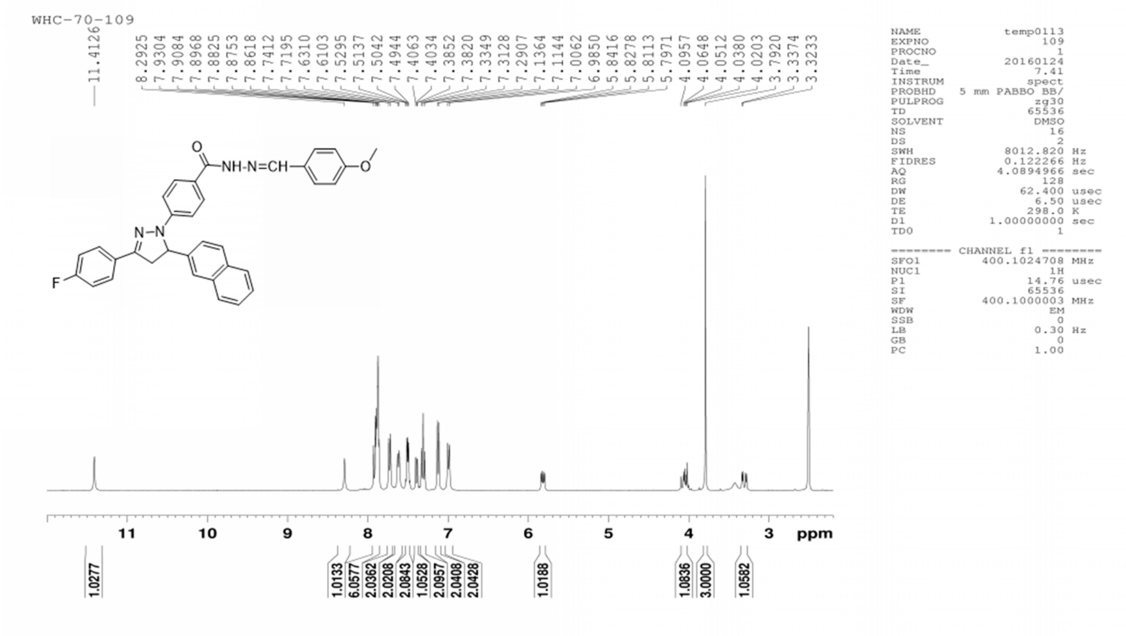

Figure S27. H27-¹H-NMR.

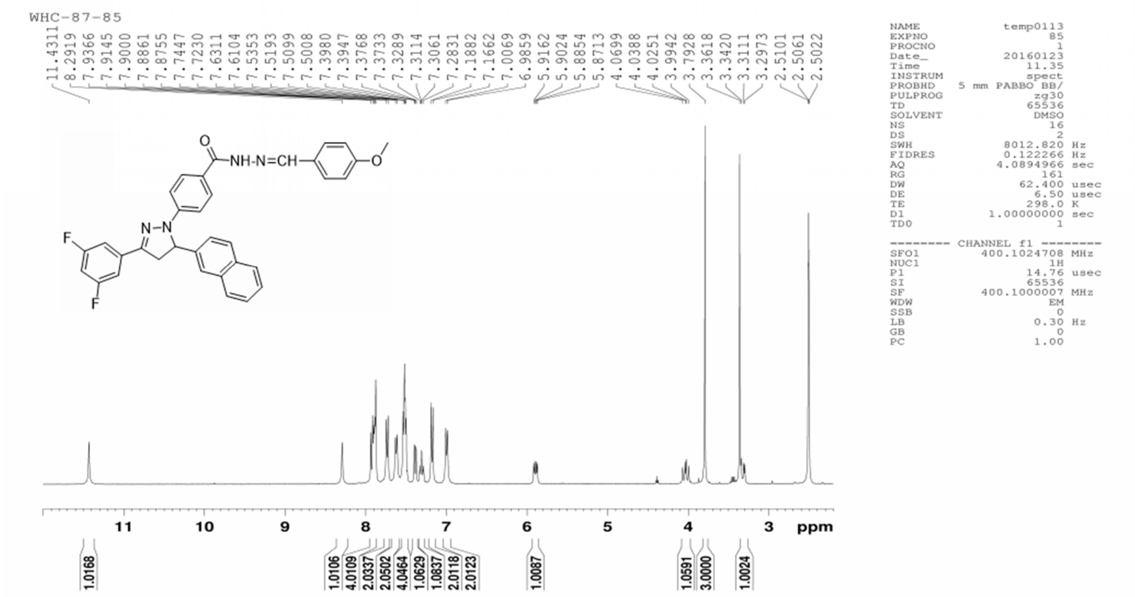

Figure S28. H28-¹H-NMR.

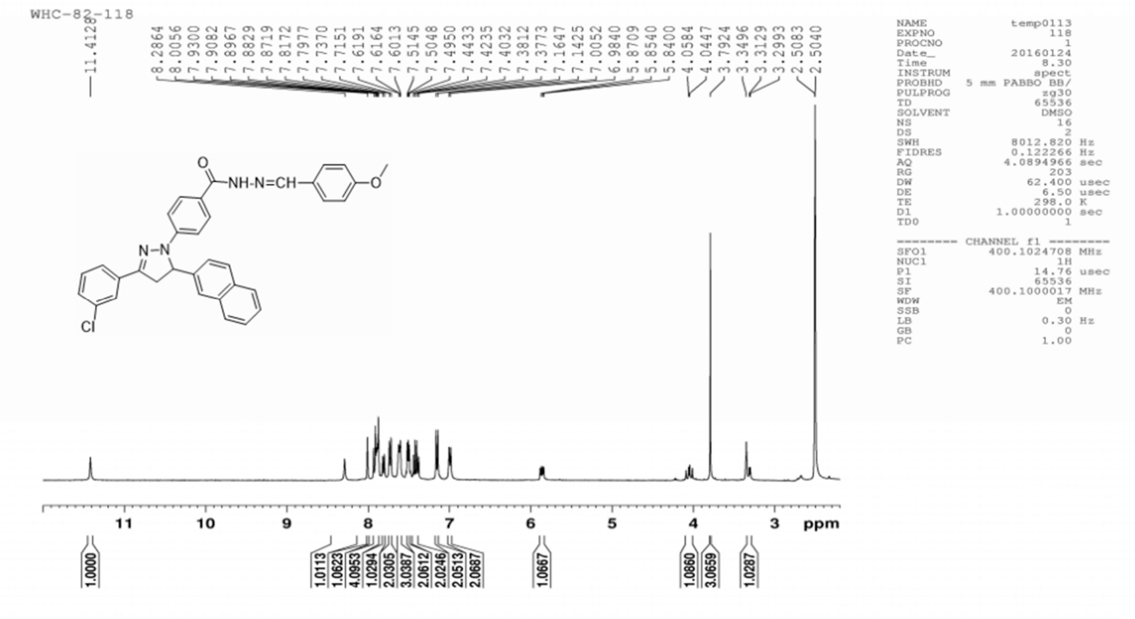

Figure S29. H29-^{1}H-NMR.

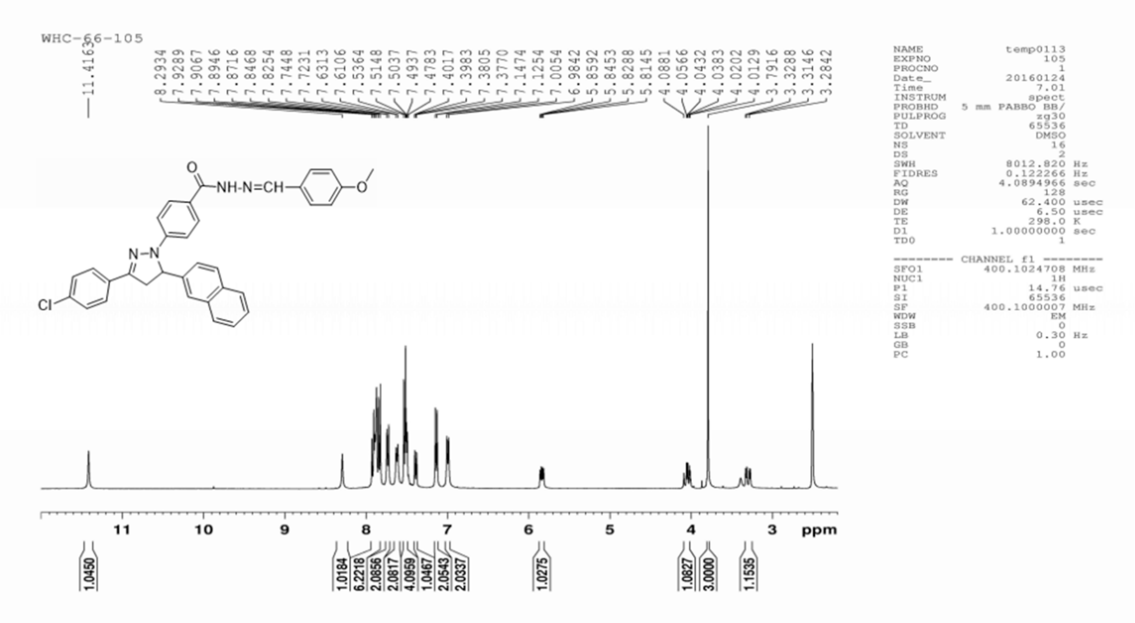

Figure S30. H30-^{1}H-NMR.

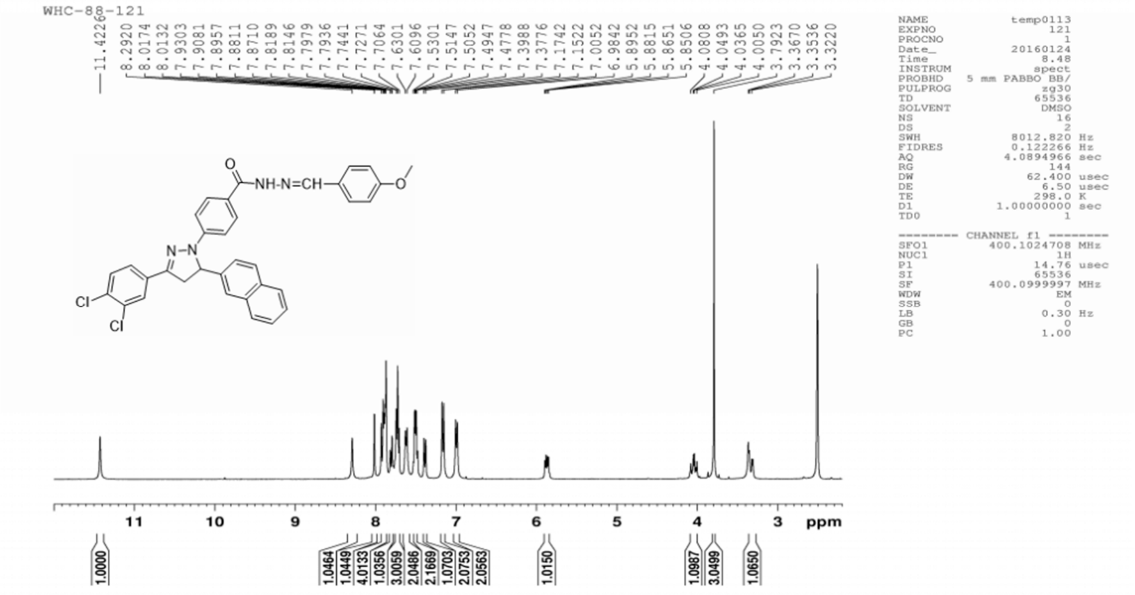

Figure S31. H31-¹H-NMR.

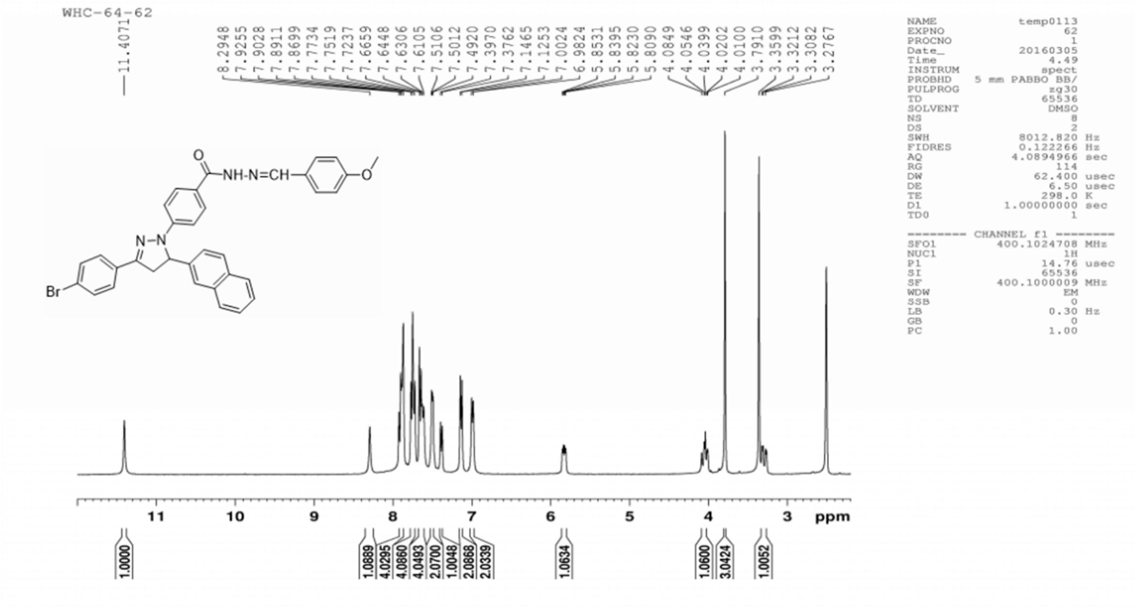

Figure S32. H32-¹H-NMR.

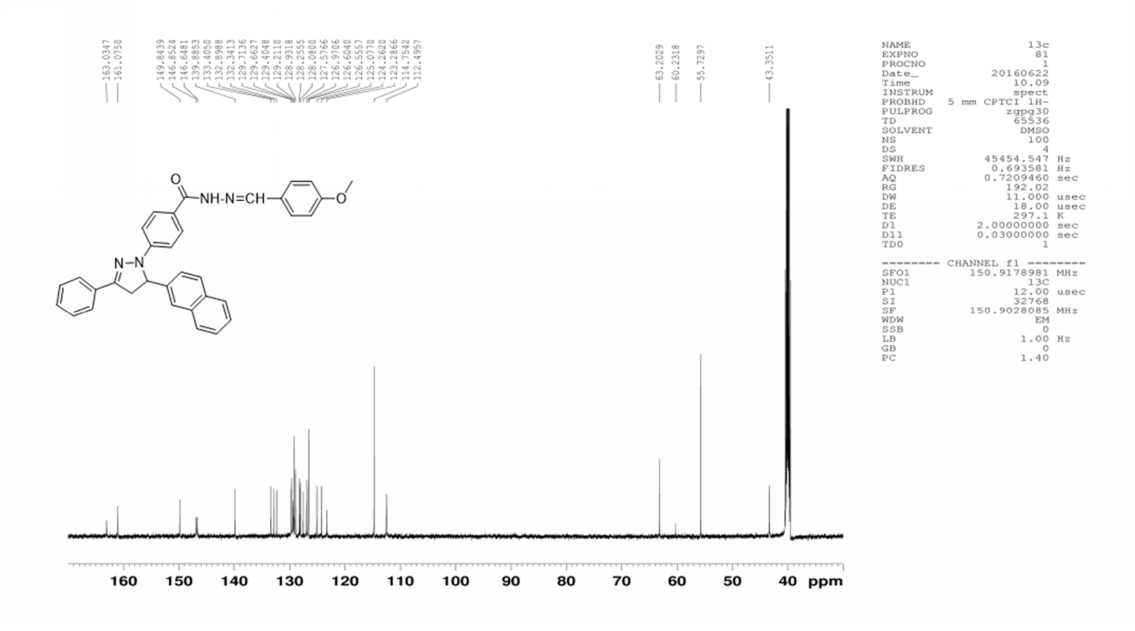

Figure S33. H17-^{13}C-NMR

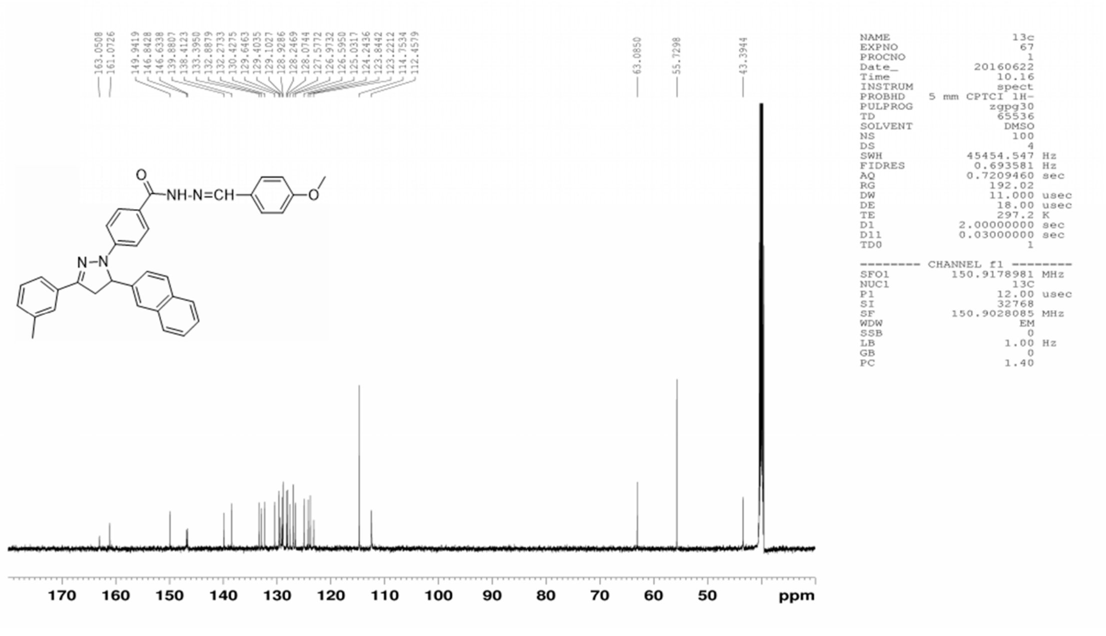

Figure S34. H19-^{13}C-NMR.

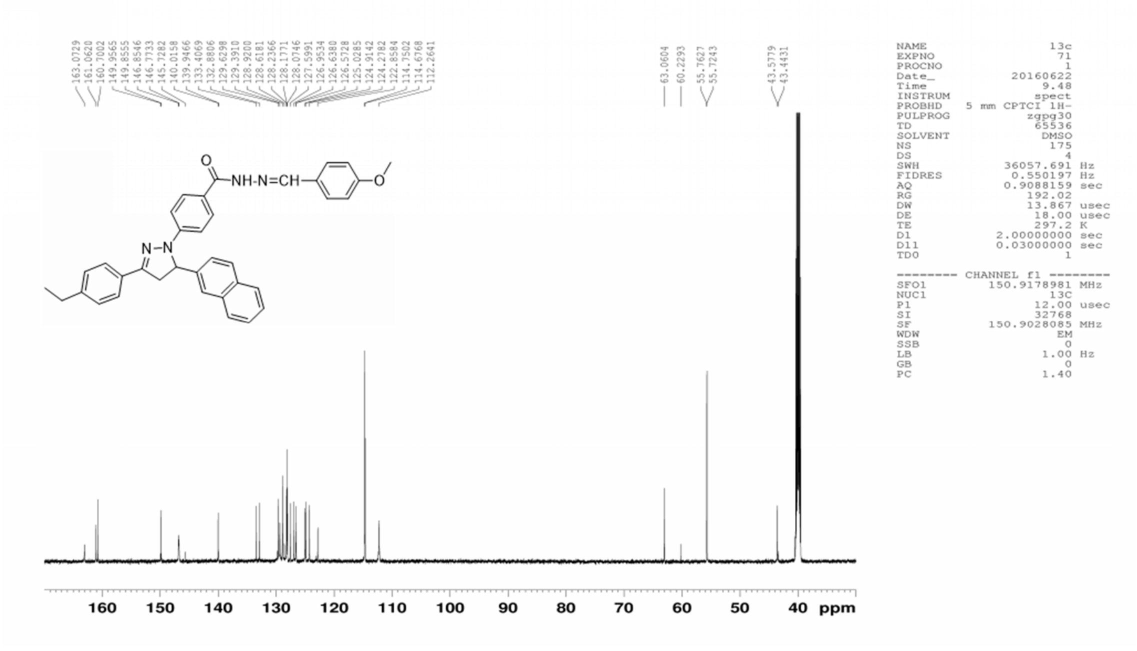

Figure S35. H21-^{13}C-NMR.

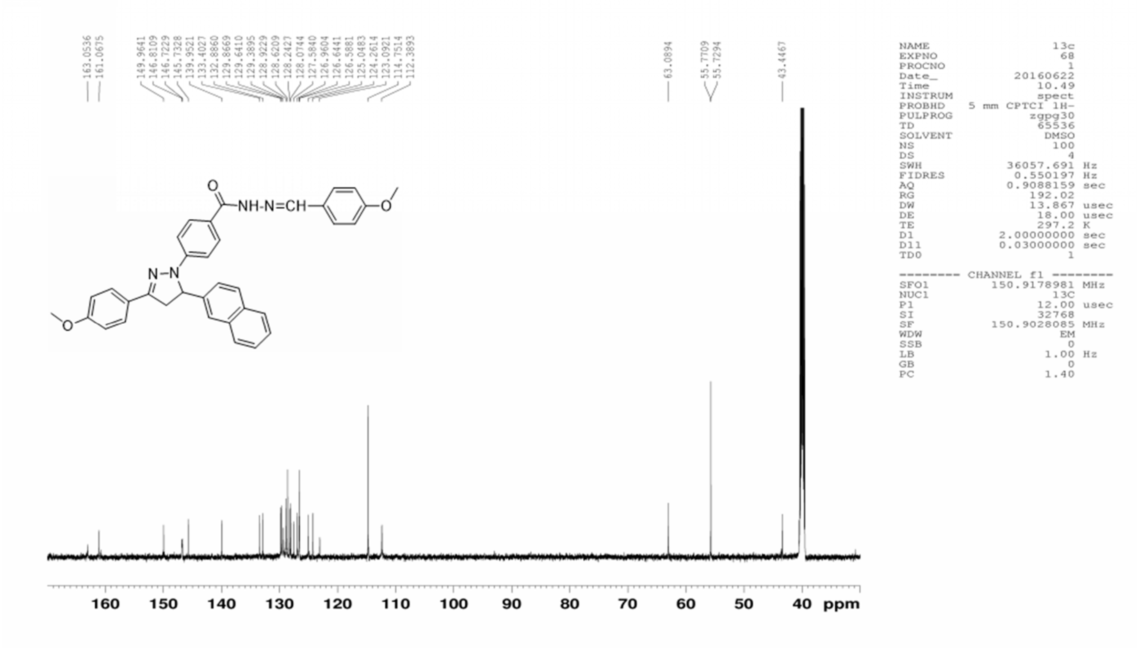

Figure S36. H22-^{13}C-NMR.

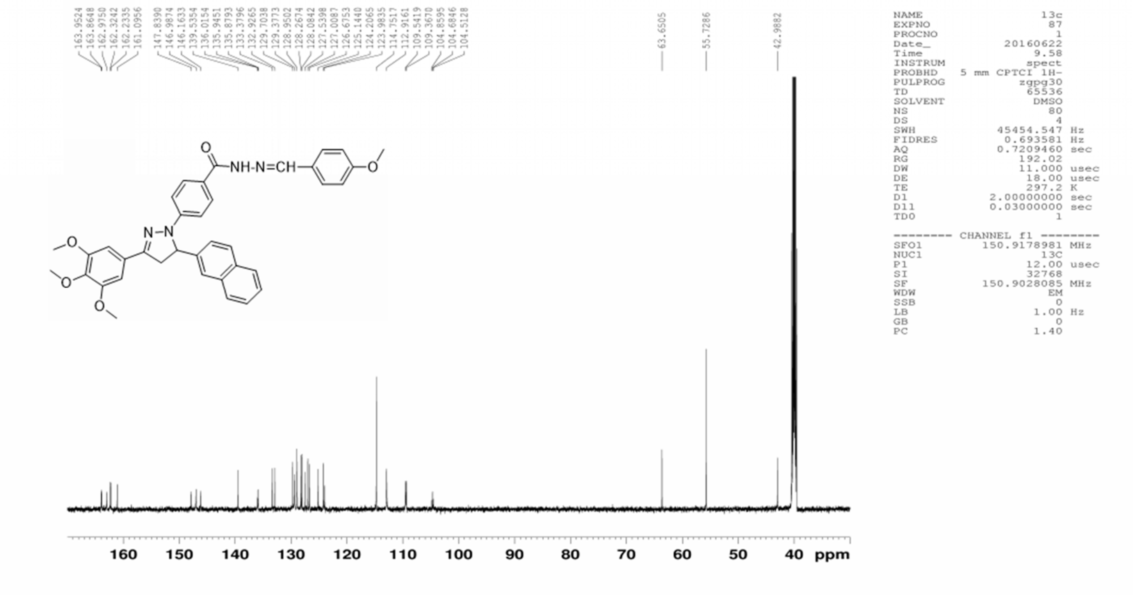

Figure S37. H23-^{13}C-NMR.

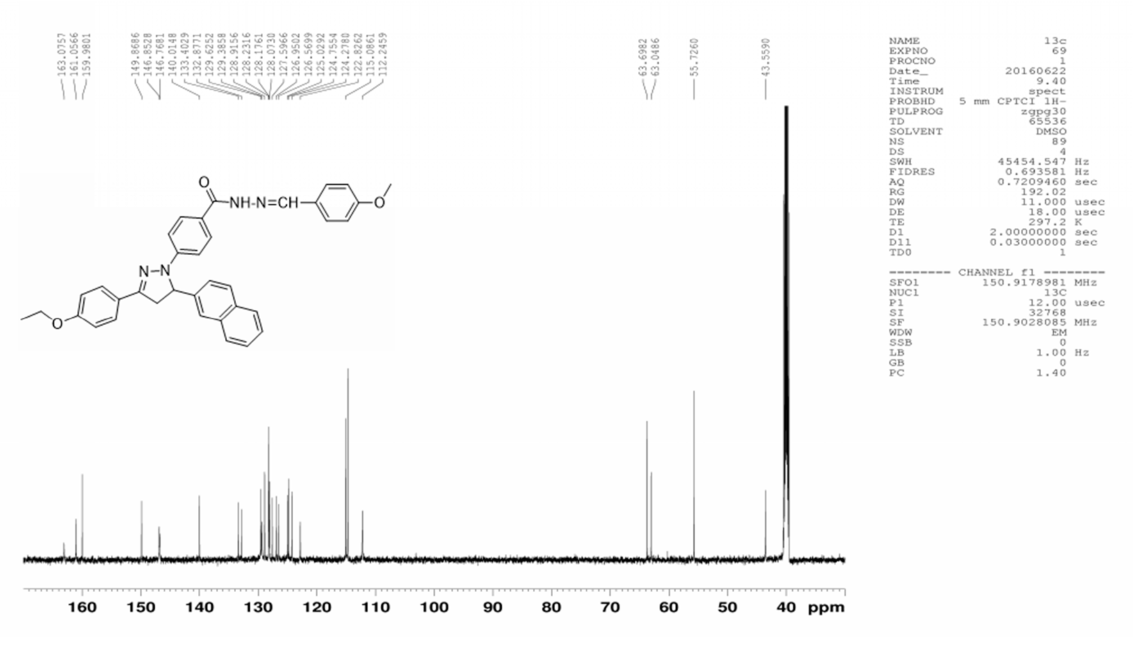

Figure S38. H24-^{13}C-NMR.

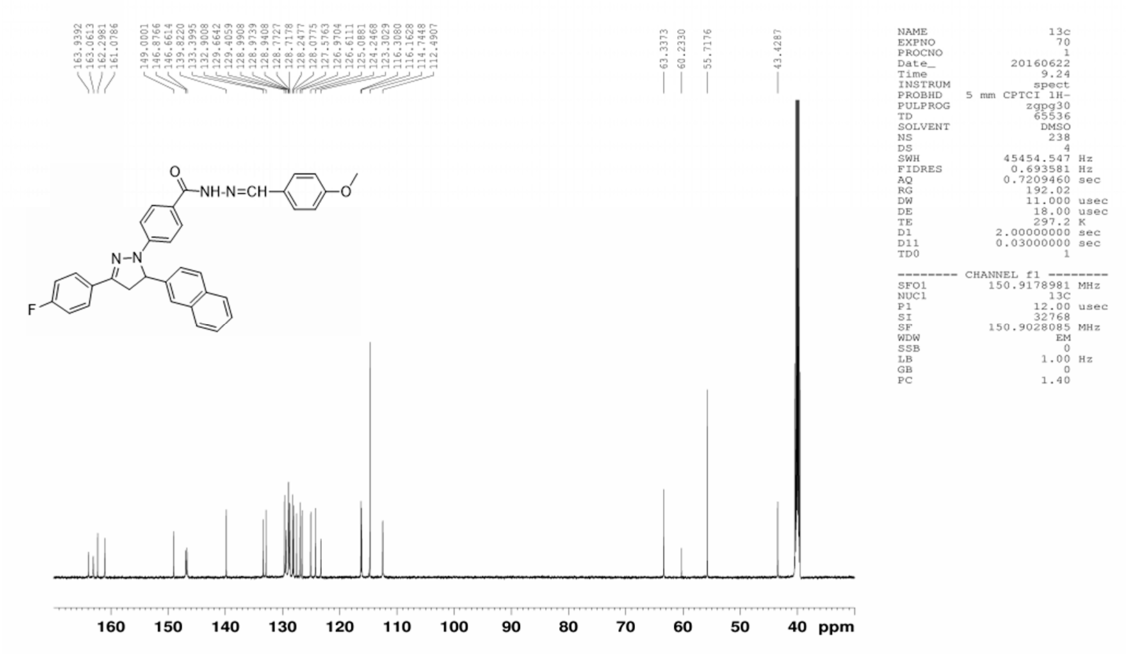

Figure S39. H27-^{13}C-NMR.

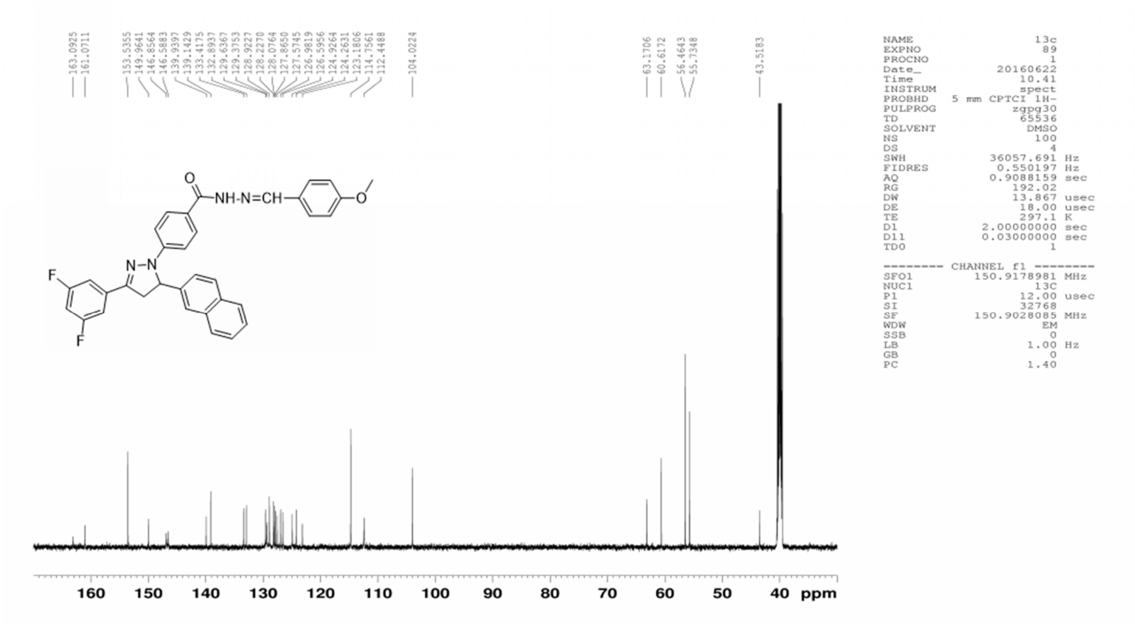

Figure S40. H28-^{13}C-NMR.

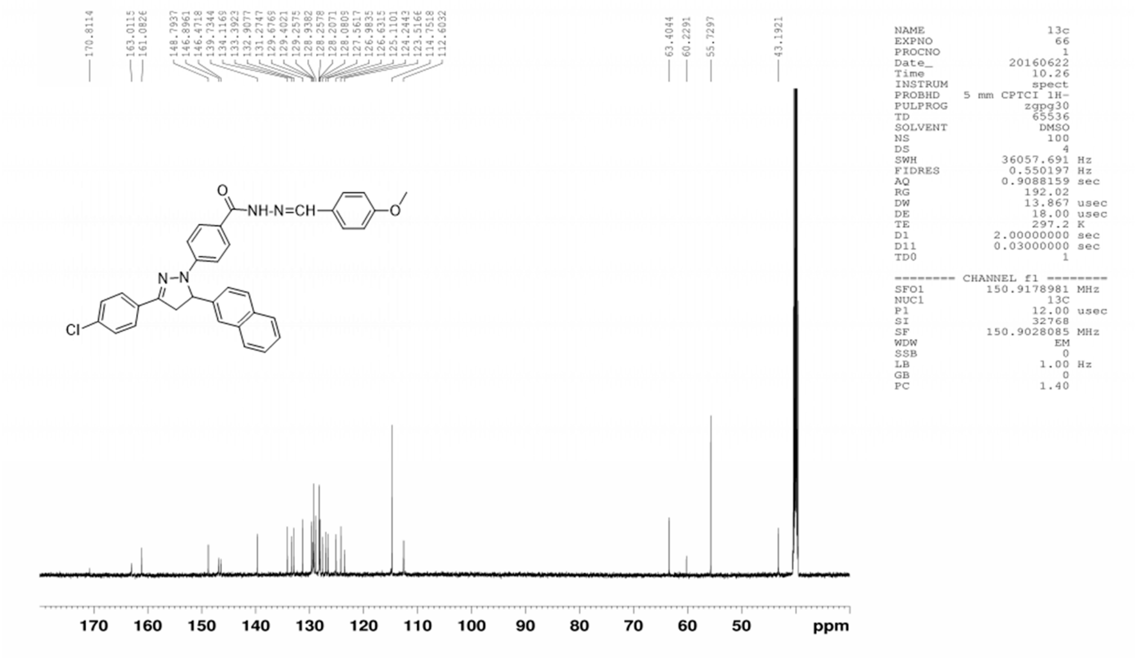

Figure S41. H30-^{13}C-NMR.

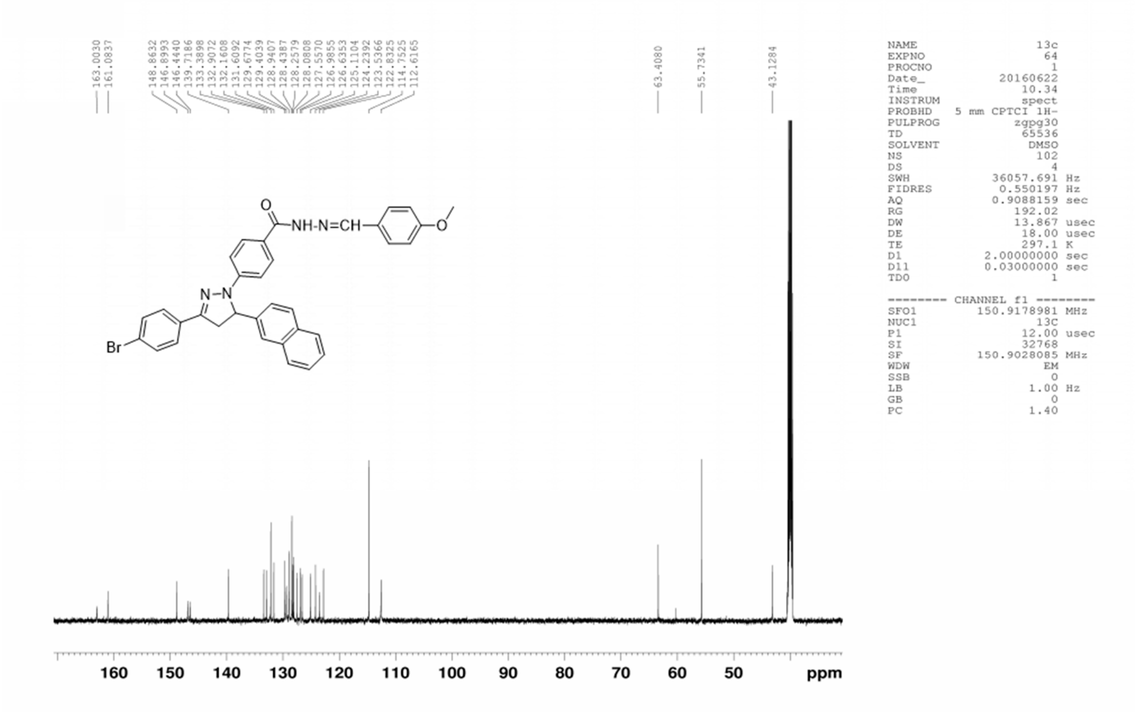

Figure S42. H32-^{13}C-NMR.
